# Low-temperature screen-printed Ag and Ag–Cu metallization for perovskite/silicon tandem solar cells

**DOI:** 10.1039/d6el00152a

**Published:** 2026-10-30

**Authors:** Kerem Artuk, Michele De Bastiani, Lisa Champault, Jun Zhao, Julien Hurni, Nicolas Badel, Antoine Descoeudres, Jean-David Decoppet, Adrien Theytaz, Jonas Geissbühler, Laurie-Lou Senaud, Vanessa Gainche, Patrick Wyss, Philippe Wyss, Florent Sahli, Ricardo Augusto Zanotto Razera, Deniz Turkay, Christian M. Wolff, Tonio Buonassisi, Christophe Ballif, Bertrand Paviet-Salomon, Quentin Jeangros

**Affiliations:** a Centre Suisse d’Electronique et de Microtechnique (CSEM) Rue Jaquet-Droz 1 Neuchâtel CH-2000 Switzerland kerem.artuk@csem.ch quentin.jeangros@csem.ch; b École Polytechnique Fédérale de Lausanne (EPFL), Institute of Electrical and Microengineering (IEM), Photovoltaics and Thin-Film Electronics Laboratory (PV-Lab) Rue de la Maladière 71b Neuchâtel 2000 Switzerland

## Abstract

We demonstrate monolithic perovskite/silicon tandem solar cells incorporating a screen-printed silver front metallization grid, achieving in-house efficiencies of up to 34.02% on a 1 cm^2^ active area, 34.01% on 4 cm^2^, and 31.29% on 54 cm^2^. Careful selection of metal pastes and optimization of the metal–ITO interface enable screen-printed electrodes to outperform evaporated Ag at the 1 cm^2^ scale while supporting scale-up to 54 cm^2^ with ∼8% relative efficiency loss. Furthermore, a proof-of-concept glass–glass encapsulated module comprising two parallel strings of two series-connected 54 cm^2^ cells (designated illumination area of 216 cm^2^) demonstrates a power conversion efficiency of 26.7% with a *V*_oc_ approaching 3.85 V. In parallel, Ag (35 wt%)–Cu pastes are shown to result in comparable tandem cell peak efficiencies, with average relative losses of ∼5% compared to Ag, offering a route to reduced silver consumption in tandems.

## Abstract 2

Screen-printed Ag metallization on ITO enables perovskite/silicon tandems of 34.0% on 1 and 4 cm^2^ and of 31.3% on 54 cm^2^, while Ag–Cu pastes cut silver use at only a minor efficiency loss.

## Graphical abstract

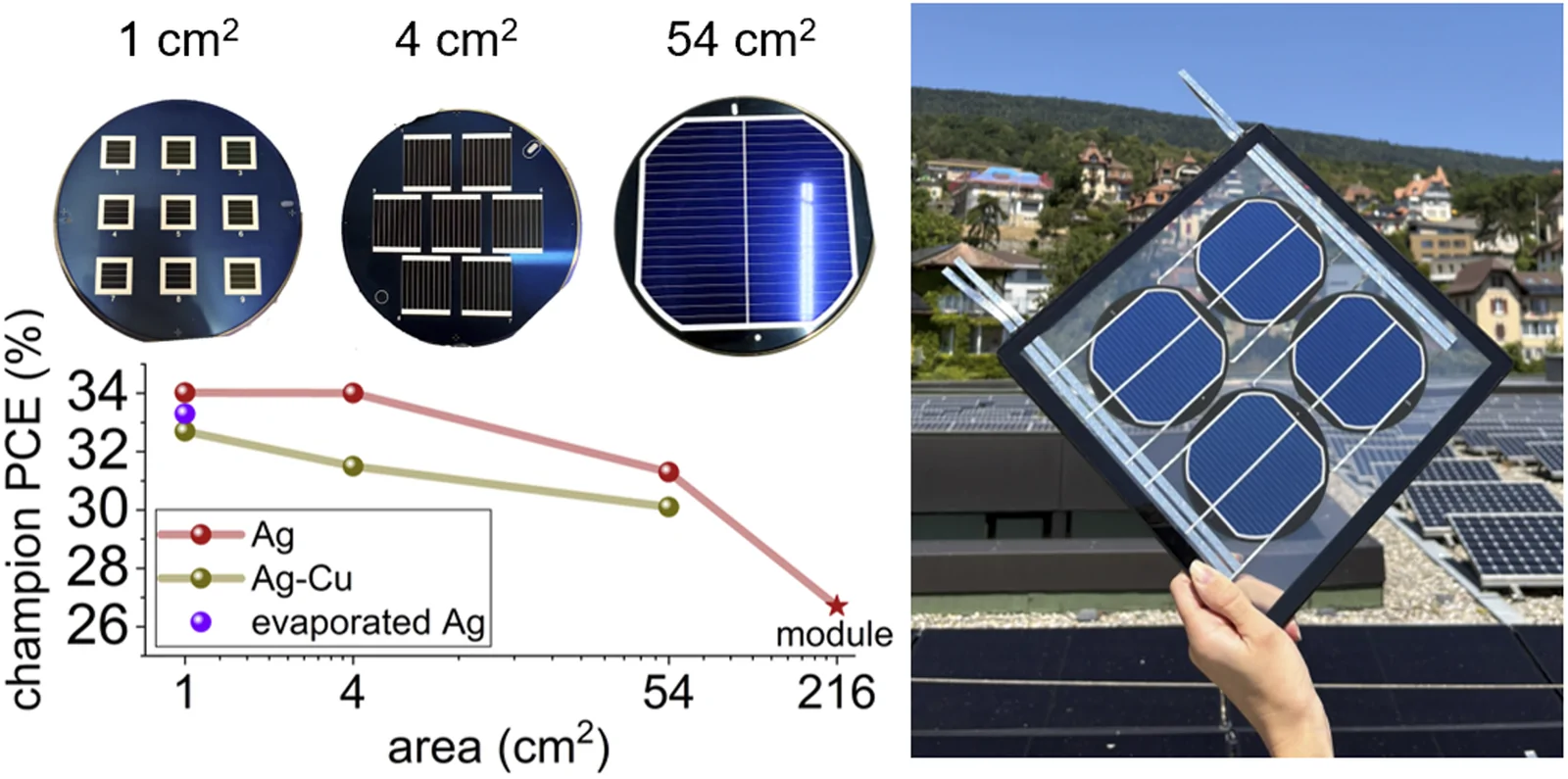

**Broader context**

Perovskite/silicon tandem solar cells are widely regarded as the most promising photovoltaic technology for surpassing the efficiency limits of single-junction silicon and accelerating the deployment of renewable electricity. However, translating laboratory-scale efficiency records into commercial products requires manufacturing processes that are scalable, cost-effective, resource-efficient, and compatible with existing photovoltaic production lines. Metallization remains a critical bottleneck, as state-of-the-art tandem devices typically rely on thermally evaporated silver grids, a fabrication approach that is inapplicable at industrial scale. In this work, we demonstrate industry-compatible screen-printed Ag and Ag–Cu electrodes for monolithic perovskite/silicon tandem solar cells, achieving efficiencies comparable to those of laboratory-scale devices while substantially reducing silver consumption through Ag–Cu metallization. By combining high performance with scalable manufacturing and lower critical material demand, this work helps bridge the gap between laboratory demonstrations and commercial deployment, contributing to more sustainable and cost-effective next-generation solar electricity generation.

Monolithic perovskite/silicon tandem solar cells have reached power conversion efficiencies (PCE) above 35% on active areas of 1 cm^2^, highlighting their high potential for next-generation photovoltaics.^1^ However, these record performances are typically achieved using an evaporated metallization grid and transparent conductive oxide (TCO) compounds that are not widely adopted in industrial manufacturing, limiting the scalability and commercial relevance of these small-area prototypes.^2,3^

Achieving high efficiencies with screen-printed metallization and industry-standard indium tin oxide (ITO), the reference TCO used in silicon heterojunction solar cells, has remained a challenge. Publicly available reports of perovskite/silicon tandem solar cells incorporating screen-printed metallization remain scarce, with efficiencies shown to reach 29.8% on a 260 cm^2^ device.^4–6^

Here, we demonstrate monolithic two-terminal perovskite/silicon tandem solar cells with active areas of 1, 4, and 54 cm^2^ incorporating a front metallization grid (Fig. 1a and b) screen-printed on ITO. The devices are fabricated on double-sided sub-micron textured n-type float-zone (FZ) or Czochralski (Cz) silicon wafers. These comprise a conventional p–i–n perovskite top cell with a spin-coated perovskite absorber deposited onto a silicon heterojunction (SHJ) bottom cell (Fig. 1c).^4^

**Fig. 1:**
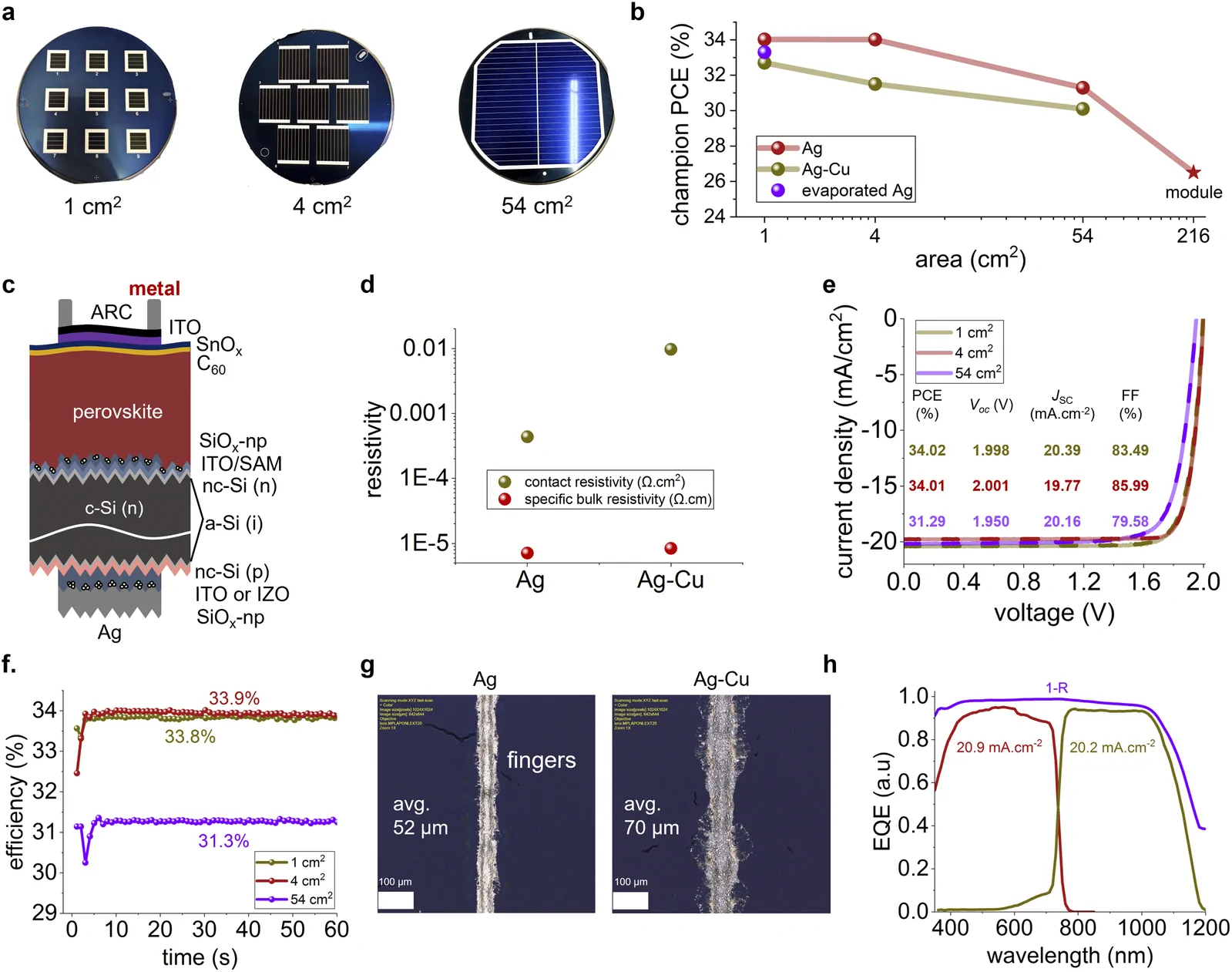
Performance of screen-printed perovskite–silicon tandem solar cells. (a) Photographs of monolithic tandem devices with active areas of 1, 4, and 54 cm^2^ fabricated on 4-inch wafers. (b) Champion reverse-scan power conversion efficiency (PCE) as a function of device area featuring either screen-printed Ag, screen-printed Ag–Cu, or evaporated Ag electrodes. (c) Schematic device architecture illustrating the perovskite–silicon tandem stack used here. (d) Extracted contact resistivity (Ω cm^2^) and bulk resistivity (Ω cm) of Ag and Ag–Cu pastes. (e) Champion reverse and forward (dashed) *JV* curves of devices fabricated with a screen-printed Ag metallization grid, and reverse-scan performance parameters. (f) Maximum power point tracking of champion cells of each active area. (g) Optical microscopy images of printed fingers showing reduced line width for Ag (∼52 µm) compared to Ag–Cu (∼70 µm). (h) External quantum efficiency (EQE) and 1-reflectance of a representative champion Ag-based device measured over its full active area, including shadowing from fingers.

As the device area increases, metallization-related resistive losses become a possible dominant performance-loss mechanism, making both the paste composition and the metal–TCO contact properties critical design parameters. To address this challenge, we screened eight commercially available metallization pastes with Ag contents (metal fraction) ranging from 30% to 100% (Fig. S1) and identified two promising candidates: an Ag paste and an Ag (35 wt%)–Cu paste. Both formulations exhibit low contact resistivity to ITO and favorable specific bulk resistivity (Fig. 1d), while remaining compatible with low-temperature processing. However, their optical and electrical characteristics differ, resulting in distinct impacts on tandem solar cell performance as discussed below.

1 cm^2^ devices with Ag screen-printed metallization achieve up to 34.02% reverse-scan efficiency, and a maximum-power-point (MPP) tracked efficiency of 33.80% (Fig. 1e and f). A sister 1 cm^2^ device was independently certified by SIMIT, achieving a reverse-scan efficiency of 32.86% and a stabilized MPP efficiency (after 5 min of tracking) of 32.21%, with a *V*_oc_ of 1.994 V (Fig. S2).

The champion Ag-based 4 cm^2^ device reaches a reverse-scan efficiency of 34.01% and a stabilized MPP efficiency of 33.90%, with the stabilized efficiency surpassing that of the best 1 cm^2^ device (Fig. 1e and f). The *V*_oc_ exceeds 2.0 V and is slightly higher than that of the 1 cm^2^ device, mainly due to an increase in the bottom-cell *V*_oc_ upon scaling.^7^ A sister 4 cm^2^ device was independently certified by ISFH, achieving a stabilized MPP efficiency (after 10 min of tracking) of 31.56%, with a *V*_oc_ of 1.982 V (Fig. S3 and S4). High fill factors approaching 86% are measured with 4 cm^2^ devices thanks to low resistive losses and a current mismatch of ∼1.5 mA cm^−2^ in favor of the perovskite top cell. Light-intensity-dependent current–voltage (*JV*) measurements reveal a series resistance of ∼2.3 Ω cm^2^ for the Ag paste device (extracted at current-matched conditions),^8^ while the pseudo fill factor reaches 86.4%, surpassing that of the 1 cm^2^ device (85.3%, see Fig. S5). This improvement is driven primarily by an increase in the silicon bottom-cell pseudo-fill factor upon scaling to 4 cm^2^ and by the associated reduction in perimeter recombination losses.^7^ These results confirm that resistive losses associated with the metal front grid remain minimal at the 1 and 4 cm^2^ scale. These high efficiencies are not limited to FZ substrates. Champion 4 cm^2^ devices fabricated on Cz wafers achieve efficiencies of up to 33.8% with an average of 33.3%, albeit using thick (∼250 µm) Cz wafers (see Fig. S6).

To assess scalability, 54 cm^2^ tandem devices were fabricated using an Ag screen-printed front grid, yielding efficiencies of up to 31.29% (reverse scan) and an MPP efficiency of 31.30% (see Fig. 1e and f). For these large-area devices, a double-print metallization strategy was employed to reduce finger line resistance and improve fill factors (see SI and Fig. S7 and S8).

Replacing Ag with an Ag–Cu paste containing 35 wt% Ag led to relative efficiency losses of approximately 4%, 7%, and 4% on 1, 4, and 54 cm^2^, respectively. While a dedicated batch demonstrated that co-processed Ag and Ag–Cu tandems can achieve comparable peak efficiencies (see Fig. S9), the Ag-based devices exhibited slightly higher average efficiency. The larger spread observed for the Ag–Cu devices is unlikely to originate from the metallization step itself and more likely reflects variations arising from preceding fabrication processes, particularly the perovskite deposition. The superior performance of the Ag paste originates from coupled electrical and optical effects. In addition to its lower contact resistivity with ITO (Fig. 1d), the paste forms narrower and more homogeneous fingers, likely due to improved printability (Fig. 1g), reducing optical shading. As a result, the champion Ag-based device reaches a summed integrated subcell photocurrent density – including shading losses from metallization – of 41.1 mA cm^−2^ (Fig. 1h). This printing behavior is not only influenced by paste chemistry but also by surface morphology: on the effectively planarized tandem front surface, resulting from the non-conformal perovskite coating (see Fig. S10), paste rheology governs finger spreading, whereas on textured silicon surfaces this effect is largely suppressed (see Fig. S11).

As most record-setting perovskite/silicon tandem solar cells rely on thin indium zinc oxide (IZO) as the top TCO,^2,3,9^ we investigated the influence of TCO composition, thickness, and metallization curing temperature on the metal–TCO contact resistance. First, reducing the curing temperature from 210 °C, the standard for silicon heterojunction solar cells, to 130 °C, for compatibility with the tandem thermal budget, resulted in only a negligible change in contact resistivity (Fig. S12). Second, decreasing the ITO thickness led to an increase in contact resistance. Third, replacing ITO with IZO while maintaining the same Ag metallization increased series resistance (extracted at tandem device level, see Fig. S13) by approximately 0.25–0.3 Ω cm^2^. This increase is likely related to the higher contact resistance measured at the metal–IZO interface, as evidenced by the contact resistivity measurements and device data shown in Fig. S12–S14, respectively.

An IZO/ITO bilayer mitigates this additional resistive loss, possibly by reducing contact resistance at the metal–TCO interface to the level of bare ITO, resulting in device performance and resistive losses comparable to ITO-only devices (Fig. S12–S15). While the more transparent IZO layer provides a slight increase in short-circuit current, this benefit is largely offset by a reduction in current mismatch, reducing fill factors. The overall gain is therefore modest, as the hydrogenated ITO used in this work already exhibits suitable optoelectronic properties. The optoelectronic properties of the various TCOs investigated in this study are summarized in Table S1.

To benchmark the effectiveness of screen-printed metallization against Ag evaporation, we compared 1 cm^2^ tandem cells fabricated on 4-inch wafers with reference devices employing evaporated Ag electrodes fabricated on 1-inch substrates (Fig. 1b and S16–S18). While the evaporated-electrode devices achieve average efficiencies of approximately 32%, with a champion efficiency of ∼33.30% (Fig. S16 and S18), the screen-printed devices reach a champion efficiency of 34.02% and an average efficiency of 33.25% (Fig. S17). In the present process comparison, the higher performance of the screen-printed devices is associated with reduced resistive losses, with a decrease in series resistance of approximately 1.0 Ω cm^2^ translating into an absolute fill factor gain of about 1.0%. This reduction originates from the ∼40-fold thicker Ag screen-printed metallization and the improved front-grid design, which together enhance current collection and lower transport losses. Notably, both approaches yield comparable *V*_oc_ values, indicating that the screen-printing and subsequent curing process do not adversely affect the top-cell quality. Optical microscopy reveals that the screen-printed Ag contacts exhibit a sharper and more homogeneous finger geometry with an average width of approximately 52 µm, compared with 60 µm for evaporated-Ag electrodes (Fig. 1g and S11). The resulting reduction in optical shading is estimated to increase photocurrent by only ∼0.05 mA cm^−2^, indicating that the impact of the metallization geometry on light harvesting is small.

We then demonstrate the integration in a module of four Ag-metallized tandem cells, each of 54 cm^2^ and connected in 2 parallel strings of 2 series-connected cells, yielding an active area of 216 cm^2^. The cells were encapsulated in glass–glass architecture laminated at ∼130 °C (see Fig. S19). The resulting proof-of-concept module achieves a power conversion efficiency of 26.7% (designated illumination area) with an open-circuit voltage approaching 3.85 V, corresponding to an average cell voltage of ∼1.925 V (see Fig. S20 for *JV* and MPP). The module performance is achieved without optical optimization, as the antireflection coating was implemented on the individual cells after TCO–metal deposition rather than on the module glass, introducing additional optical losses. In particular, placing the antireflection coating beneath the encapsulation stack disrupts the intended refractive-index matching.

Finally, the reliability of Ag and Ag–Cu tandems was evaluated to assess the impact of the metal grid composition (Fig. 2a and b). Ag and Ag–Cu 1 cm^2^ screen-printed tandem devices without perovskite/C_60_ interface passivation (see Table S2 and Fig. S21 for details) were encapsulated in a glass/glass/lamination film stack using an edge sealant. Device performance statistics measured after encapsulation are provided in Fig. S22. Ag devices exhibited slightly higher *J*_sc_, likely due to improved printability and sharper finger definition (see Fig. S11), and lower series resistance and a correspondingly higher FF due to lower contact resistance with the ITO. Consequently, Ag devices exhibited a slight efficiency advantage, delivering an average PCE approximately 0.5%_abs_ higher than that of Ag–Cu devices in this batch.

**Fig. 2:**
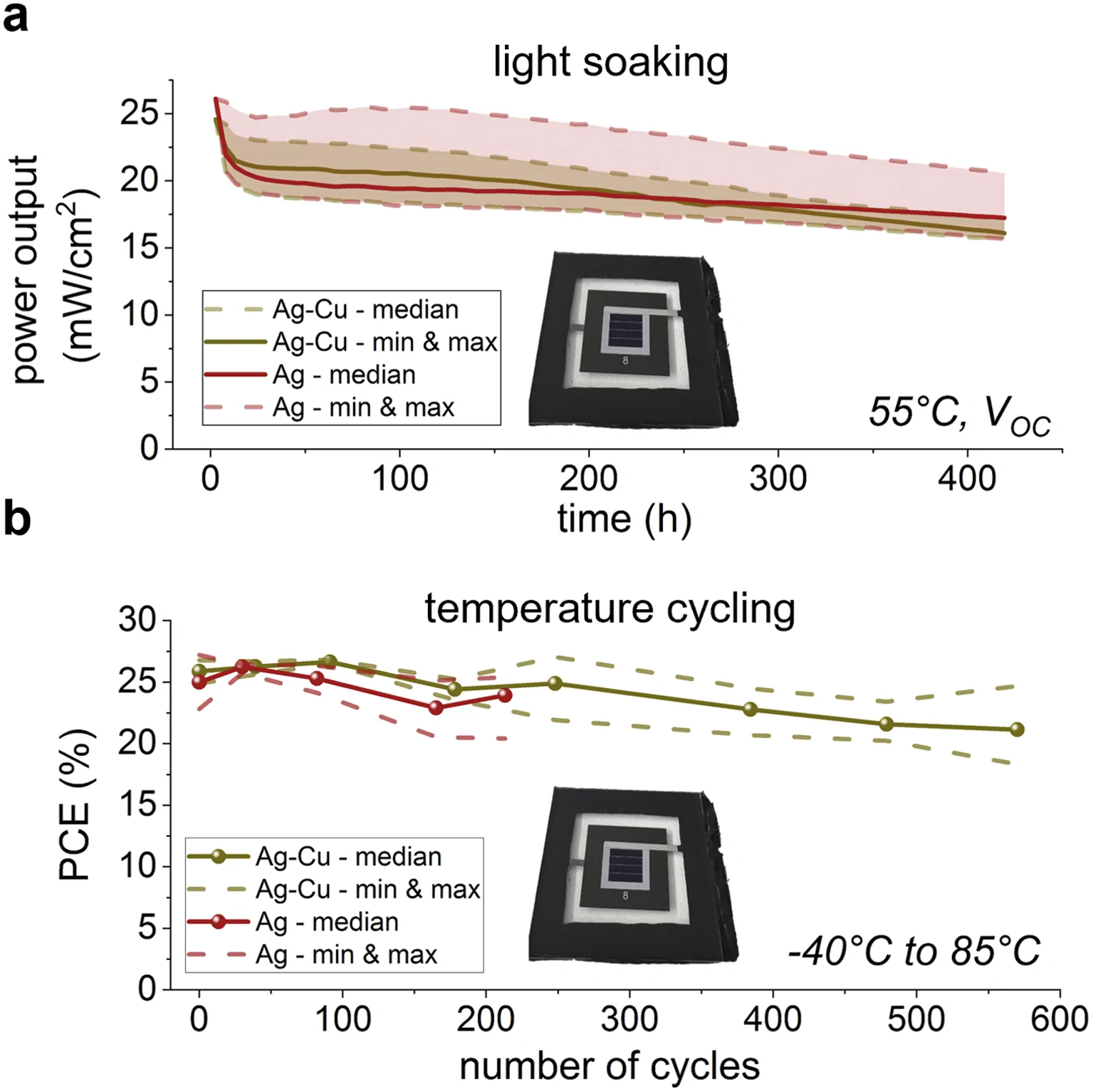
Stability assessment of screen-printed 1 cm^2^ perovskite–silicon tandem solar cells employing Ag and Ag–Cu metallization (without perovskite/C_60_ interface passivation). (a) Light-soaking stability at 55 °C under *V*_oc_ conditions (*JV* curves measured every 15 min), showing the evolution of power output (mW cm^−2^) over 420 h for Ag (*N* = 8) and Ag–Cu (*N* = 8) devices. Solid lines represent median values and dashed lines indicate the minimum–maximum range; (b) thermal cycling performance (from −40 to 85 °C, IEC 61215), displaying PCE evolution as a function of cycle number.

The encapsulated cells were subsequently subjected to light soaking at open-circuit under 1-sun illumination at 55 °C. Both Ag and Ag–Cu devices exhibited comparable initial power outputs of approximately 25–26 mW cm^−2^ and degraded progressively during ∼420 h of aging, ultimately converging to similar final values of ∼16–18 mW cm^−2^ (Fig. 2a). While both populations exhibited a similar initial burn-in behavior and reached comparable final performance, a linear fit over the final 200 h of the tests revealed a somewhat faster degradation rate for the Ag–Cu devices (−0.052 *vs.* −0.028% h^−1^ in PCE). This difference originated primarily from FF and *J*_sc_ losses, while *V*_oc_ remained more stable for both groups of cells (Fig. 2a, S23 and S24). The origin of this behavior could be explained by enhanced ionic losses;^10,11^ however, confirming whether such a degradation mechanism is related to the presence of Cu in the metallization grid is beyond the scope of this manuscript.

Furthermore, during thermal cycling from −40 to 85 °C using an IEC 61215-inspired temperature profile (Fig. 2b), both metallization systems exhibited ∼4% relative degradation after one qualification sequence (TC200), indicating comparable performance under the applied thermomechanical stress conditions. Thermal cycling was subsequently extended for the Ag–Cu devices, which retained 86% of their initial average power conversion efficiency after 600 cycles (TC600). Lastly, under damp-heat exposure (85 °C, 85% RH) (Fig. S25), the Ag-based devices retained 94.7% of their initial average power conversion efficiency after 1000 h.

In conclusion, we demonstrated monolithic perovskite/silicon tandem solar cells with efficiencies of 34.02%, 34.01%, and 31.29% on active areas of 1, 4, and 54 cm^2^, respectively, using screen-printed front metallization on an industry-standard ITO transparent conductive oxide. These results highlight the importance of the metallization and metal–TCO interface optimization to enable efficient scaling of tandem solar cells. Devices employing the optimized screen-printed Ag grid achieved higher efficiencies than the evaporated-reference devices used in this study at the 1 cm^2^ scale. Reduced-silver Ag–Cu pastes offer a promising pathway toward lower Ag consumption, although additional optimization of paste formulation, contact formation, and printing processes will be required to close the small 5% relative performance gap with Ag. Finally, the reliability of tandem devices with Ag and Ag–Cu metallization was evaluated. Thermal-cycling tests revealed similar degradation behavior for both paste formulations. Under light soaking, Ag–Cu devices exhibited a slightly faster degradation trend; however, both populations converged to comparable average PCE values by the end of the test period.

## Conflicts of interest

The authors declare no competing financial interest.

## Supplementary Material

EL-OLF-D6EL00152A-s001

## Data Availability

The data supporting this article have been included as part of the supplementary information (SI). Experimental details and additional data can be found in the SI. Supplementary information is available. See DOI: https://doi.org/10.1039/d6el00152a.
