# Supplementary material for: Low-temperature screen-printed Ag and Ag–Cu metallization for perovskite/silicon tandem solar cells
Source: EES Solar. 2026 Oct 30. Online ahead of print. doi: 10.1039/d6el00152a (PMC13749208; doi:10.1039/d6el00152a)
Supplement: Supplementary file 1 [file EL-OLF-D6EL00152A-s001.pdf]

Supplementary Information (SI) for EES Solar.
This journal is © The Royal Society of Chemistry

Supplementary Information

Low-temperature screen-printed Ag and Ag-Cu metallization for perovskite/silicon tandem solar cells

Artuk *et al.*

Corresponding authors: Kerem Artuk, kerem.artuk@csem.ch; Quentin Jeangros, quentin.jeangros@csem.ch

Experimental Methods

Fabrication of monolithic perovskite-silicon heterojunction tandem solar cells

The bottom cell fabrication process is performed using n-type FZ Si wafers (200 µm, 2 Ω.cm) or n-type Cz (250 µm, 15 Ω.cm) Si wafers. Both sides are sub-micron textured using a KOH solution with an additive to create a random pyramid pattern, and samples undergo a cleaning sequence prior to the plasma-enhanced chemical vapor deposition (PECVD) process. Hydrogenated amorphous and nanocrystalline Si layers are deposited on both sides by PECVD. On the front side, a-Si:H(i), a-Si:H(n), and nc-Si:H(n) are deposited at 200°C. On the rear side, a-Si:H(i) is deposited at 200°C, followed by ultra-thin SiO_x and nc-Si:H(p) layers at 175°C (*1*, *2*). Then, 25 nm-thick IZO or ITO is deposited by sputtering through a shadow mask. The samples then receive a SiO_x nanoparticles (np) coating on the pyramidal textures, which acts as an infrared rear reflector (*3*). This partial layer is then capped with an 800 nm-thick Ag layer deposited by sputtering through the same shadow mask used for the TCO deposition. An additional 40 nm of NSCOT is sputtered onto the active area on top of Ag to act as a protective layer. The front side is completed by sputtering a 20 nm-thick ITO layer, which acts as recombination junction. Samples are then annealed at 210°C for 10 min in ambient conditions to recover from sputtering damage.

After an UVO treatment on the bottom-cells, a 1:1 mixture of 1 mg mL^{-1} of Me-4PACz (TCI or Dyenamo) and 4PABCz (Suzhou LiWei Tech. Co.) in EtOH:MeOH is spin-coated at 3000 rpm. The stack is then annealed at 115°C for 10 min. To improve the wetting of the perovskite precursor, 0.1 wt% SiO_x-np (~20 nm diameter) in ethanol is spin-coated statically at 3000 rpm, followed by an annealing step of 5 min at 115°C (*4*). The absorber used here is $Cs_{0.05}(FA_{0.9}MA_{0.1})_{0.95}Pb(I_{0.80}Br_{0.20})_3$ + 2% ($MAPbCl_3$, which is supplied presynthesized by Suzhou LiWei Tech. Co.). 2 mM pentafluorobenzyl phosphonic acid (pFBPA - Abcr) is added as an additive into the ink following our previous work (*5*). The molarity of the solutions is 1.63 M. A three-step spin program with 1000 rpm (10 s), 2000 rpm (30 s), and 4000 rpm (15 s) is used. With 10 s left during the spin program, 2.1 mL of Anisole antisolvent (Sigma Aldrich, >99%) is dropped onto the substrate. Then the samples are annealed at 100°C for 15 min inside a N_2 glovebox.

Prior to C_{60} deposition, the perovskite surface is passivated using one of two approaches reaching similar performance. In the first approach, a nominal 1 nm-thick $EDAI_2$ layer is thermally evaporated in an Angstrom Engineering evaporator immediately before C_{60} deposition. In the second approach, a solution containing PCl (0.075 mg mL^{-1}, Dyenamo) and 3MTPAI (0.5 mg mL^{-1}, Suzhou LiWei Tech. Co.) dissolved in a 1:1 (v:v) mixture of isopropanol (IPA) and fluorinated isopropanol (F-IPA) is spin-coated onto the perovskite surface at 6000 rpm, followed by annealing at 100 °C for 5 min (*5–8*).

Then 20 nm of C_{60} is evaporated in an Angstrom Engineering evaporator. 125 or 150 cycles (around 17 to 20 nm) of ALD-SnO_x is deposited in an Oxford FlexAl ALD system at 100°C. 35-40 nm of ITO with a sheet resistance of around 150 Ω/sq is deposited with a power of 400 W in an Octopus II system. After the TCO, ~20 µm of either Ag or Ag-Cu paste is screen-printed and cured at <130 °C. For the 54 cm^2 cells, printing is repeated twice. For the anti-reflective layer, 100 nm of NaF with 1 Å/s rate is evaporated in an Angstrom Engineering evaporator.

Single-cell encapsulation for reliability testing

Each wafer yields 9 cells of 1 cm^2, each laser-cut into 2.5 × 2.5 cm^2 coupons. The individual 1-cm^2 devices are then encapsulated using a thermoplastic polyolefin encapsulant film at temperatures lower than 140 °C in a glass-glass stack. A polyisobutylene edge sealant is applied to ensure effective moisture protection. Electrical interconnection is achieved using adhesive conductive ribbons.

Module fabrication

Four monolithic perovskite/silicon tandem solar cells with an active area of 54 cm^2 are interconnected in 2 strings of 2 cells each to form a mini module with a total active area of 216 cm^2. Electrical interconnection between the cells is achieved using conductive adhesive ribbons. The module is encapsulated in a glass-glass architecture following a procedure similar to that used for the 1-cm^2 cell encapsulation using a polyolefin encapsulant, albeit with increased lamination pressure and extended processing time to ensure uniform encapsulation of the larger-area assembly.

Before module integration, the individual cells incorporated an antireflection coating and exhibited efficiencies of approximately 30%. As a result, the optical design of the encapsulated module was not fully optimized, since the antireflection coating was deposited on the individual cells rather than on the front module glass.

Characterization

TLM stack and measurements

Contact resistivity was extracted using transfer length method (TLM) structures consisting of textured silicon wafer substrates isolated with thick PECVD-deposited SiO_x followed with a TCO layer and screen-printed metallization. The TCO was electrically isolated by laser scribing all around the TLM pad area to create appropriate isolation, followed by TLM measurements. The contact resistivity was extracted from the measured resistance as a function of contact spacing following the usual TLM method.

JV

All *JV* measurements are made on a large-area class A+A+A+ solar simulator from WACOM using two light sources (xenon and halogen). The spectral response and overall light intensity of the simulator are verified with externally calibrated reference cells to approximate 1-sun AM1.5G equivalent intensity. Initially, the intensity of the Xe lamp is adjusted using a calibrated silicon solar cell covered with a KG5 filter. The intensity of the halogen lamp is then adjusted using a calibrated silicon solar cell covered with an RG780 filter. Finally, the overall spectrum is checked using a calibrated encapsulated cell without a filter. The calibration of the Si cells (including measurements with filters) is performed by ISFH.

All measurements are performed using a four-point method. The cells are measured on a metallic vacuum chuck at a temperature set to 25°C. The active area of the devices is masked with a laser-cut aperture mask. All *JV* curves are scanned between 2.01 V and -0.02 V at a scan rate of approximately 80 mV/s. Maximum power-point tracking is achieved using a 3-point algorithm. Small areas (4 cm^2) are contacted using two Kelvin probes on either side of the metal grid. Large area cells (54 cm^2) are contacted using four Kelvin probes with one contacting each end of each bus bar. Shading due to the probe bars is corrected after the measurements by application of the correction factor (×1.007) to the J_{sc}.

EQE

The EQE of the tandem devices is measured over the full cell area, including shading from the front metallization fingers, using a PV Tools LOANA system. For top-cell EQE measurements, the voltage bias (typically 0.5–0.7 V) and infrared light bias are adjusted to maximize the response of the perovskite top cell (e.g., at 550 nm) while minimizing the contribution of the silicon bottom cell (e.g., at 1000 nm). For bottom-cell EQE measurements, a blue LED light bias and a voltage bias of typically 1.0 –1.2 V are applied to saturate the top cell and maximize the response of the silicon bottom cell.

Light soaking

For the light soaking, a simplified HTL stack is utilized with DBC (Dyenamo) as the SAM. SiO_x-np and $EDAI_2$ passivation are removed from the device stack. The ALD-SnO_x thickness was increased to 35 nm. Differences are summarized in Table S2.

Devices for light-soaking experiments are prepared by laser-cutting 1-cm^2 cells from completed 4-inch wafers featuring 9 tandems of 1 cm^2. The cells are encapsulated in a glass/glass architecture with polyolefin lamination foil and an edge sealing before testing. Aging is performed using a custom degradation system equipped with blue and infrared LEDs, providing an illumination intensity corresponding to approximately 1 sun. The sample temperature is maintained at approximately 55 °C throughout the test. Current density–voltage (*JV*) measurements are acquired every 15 min, while the devices are held under V_{oc} conditions between successive *JV* measurements.

PL imaging

Photoluminescence imaging was performed using a custom setup. Excitation of the perovskite layer is done using an array of blue light-emitting diodes (450 nm central wavelength), and effective generation is roughly equivalent to 1 sun. The sample emission is filtered using long-pass filters and imaged using a Thorlabs Kiralux CCD camera mounted with a 6 mm focal length objective. Reference for the filters in case: Thin film filter for PK PL: ThorLabs FELH-0650 and Absorbing filter for PK PL: SCHOTT RG-695 and SCHOTT RG-715.

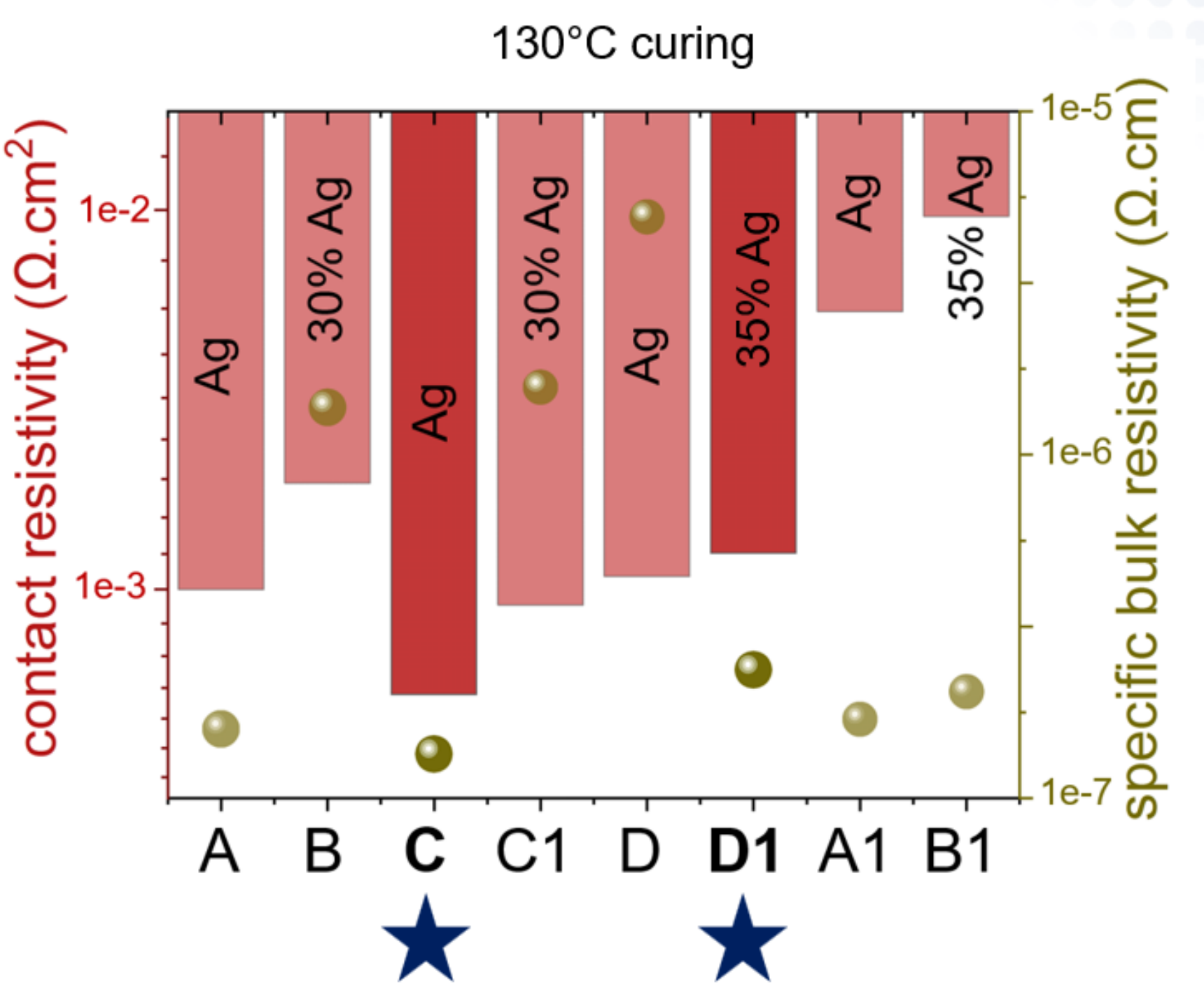

Figure S1. Contact resistivity with ITO and specific bulk resistivity for eight investigated commercial silver pastes. Identical letters indicate pastes originating from the same supplier.

中国认可
国际互认
检测
TESTING
CNAS L8490

CMA 23001716974

Test and Calibration Center of New Energy Device and Module,
Shanghai Institute of Microsystem and Information Technology,
Chinese Academy of Sciences (SIMIT)

Measurement Report

Report No. 26TR060201

**Client Name**	EPFL PV-Lab & CSEM
**Client Address**	Neuchâtel Switzerland
**Sample**	Perovskite/ silicon tandem solar cell
**Manufacturer**	EPFL PV-Lab & CSEM
**Measurement Date**	2nd June, 2026

**Performed by:**	Qiang Shi *Qiang Shi*	**Date:** *2/06/2026*
**Reviewed by:**	Wenjie Zhao *Wenjie Zhao*	**Date:** *02/06/2026*
**Approved by:**	Zhengxin Liu *Zhengxin Liu*	**Date:** *02/06/2026*

Address: No.235 Chengbei Road, Jiading, Shanghai **Post Code:** 201800
E-mail: solarcell@mail.sim.ac.cn **Tel:** +86-021-69976905

The measurement report without signature and seal are not valid.
This report shall not be reproduced, except in full, without the approval of SIMIT.

**Sample Information**	
Sample Type	Perovskite/ silicon tandem solar cell
Serial No.	257385-5#
Lab Internal No.	26060201-1#
Measurement Item	I-V characteristic
Measurement Environment	(25.7±2.0) °C, (52.1±5.0) %R.H

**Measurement of I-V characteristic**	
**Reference cell**	Abet 211458, mono-Si, WPVS,<br>calibrated by NRL (ISO tracking #: 2135)
**Calibration Value/Date of Calibration for Reference cell**	139.1 mA/ Aug. 2025
**Measurement Conditions**	Standard Test Condition (STC): AM1.5, 1000W/m^2, 25°C
**Measurement Equipment/ Date of Calibration**	AAA Steady State Solar Simulator (YSS-T155-2M) / Oct.2025<br>IV test system (ADCMT 6246) / June. 2025<br>Measuring Microscope (MF-B2017C) / July.2025<br>SR Measurement System (CEP-25ML-CAS) / May.2025
**Measurement Method**	The solar simulator was adjusted to ensure the spectral mismatch factor for each sub-cell remained within 1.000±0.015. Irradiance was set to 1 Sun based on the reference cell calibration value and spectral mismatch factor of the current-limiting junction.<br>A bidirectional logarithmic voltage sweep, from open circuit voltage (Voc) to short circuit current (Isc) and back, was performed within a single flash in accordance with IEC 60904-1:2020.<br>MPP-Tracking was conducted for 300s using the P&O method, with a sampling interval of 1 s. It was considered to have reached steady-state when the rate of change in Pmax during the final 60 seconds was less than ±0.2%/min. The reported results correspond to the average values over the last 30 seconds of the tracking period.
**Measurement Uncertainty**	Area: 1.0%(k=2); Isc: 2.3%(k=2); Voc: 1.0%(k=2);<br>Pmax: 2.7%(k=2); Eff: 2.8%(k=2).

Report No. 26TR060201

====Measurement Results ====

	Forward Scan (Isc to Voc)	Reverse Scan (Voc to Isc)	MPP-Tracking
Area		0.9492 cm^2	
Isc	19.201 mA	19.202 mA	/
Voc	1.987 V	1.994 V	/
Pmax	30.942 mW	31.189 mW	30.573 mW
Ipm	17.790 mA	17.879 mA	17.625 mA
Vpm	1.739 V	1.744 V	1.735 V
FF	81.11 %	81.44 %	/
Eff	32.60 %	32.86 %	32.21 %

- Spectral mismatch factor SMM_{top}=1.0050, SMM_{bot}=1.0070.
- Designated illumination area defined by a thin mask was measured by measuring microscope.
- MPPT results correspond to the average values over the last 30 seconds after steady-state was reached.
- Test results listed in this measurement report refer exclusively to the mentioned measured sample.
- The results apply only at the time of the test, and do not imply future performance.

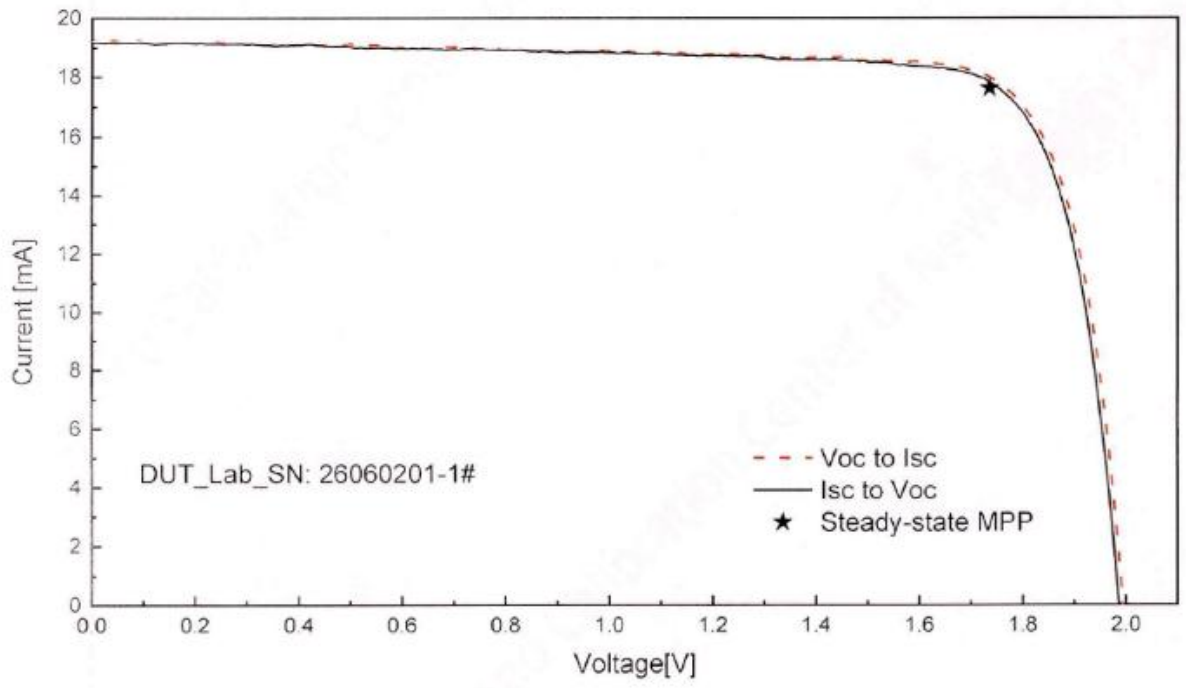

Fig.1 I-V curves of the measured sample

Report No. 26TR060201

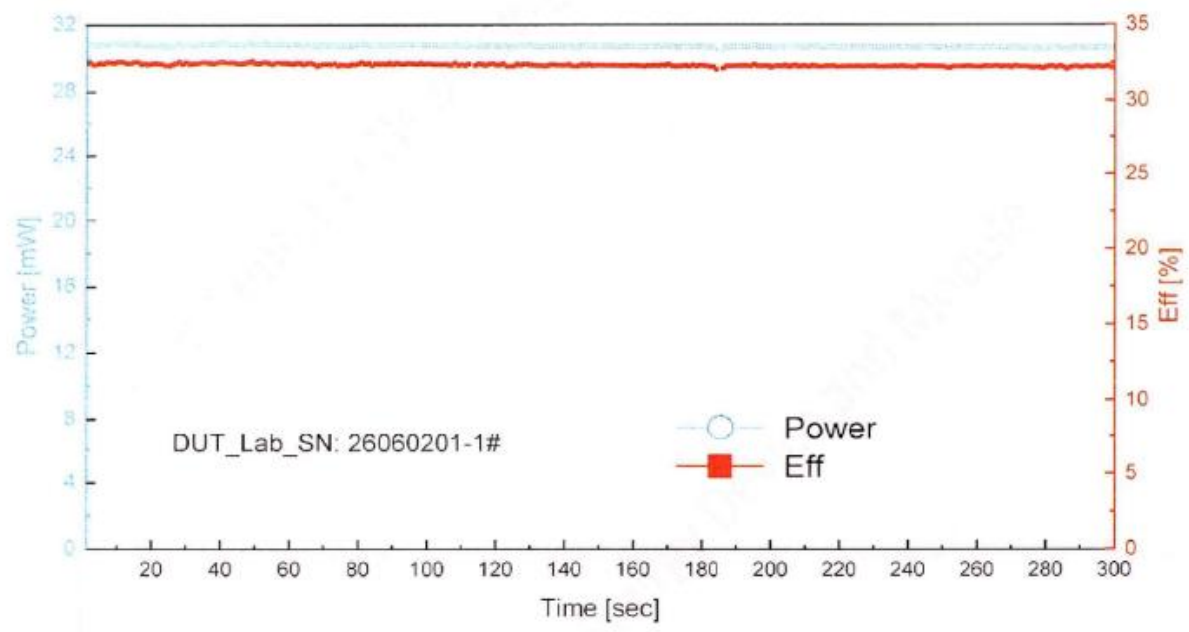

Fig.2 Steady-state maximum power output of the measured sample

-------End of Report-------

Figure S2. Certification results (*JV* and MPP data) from SIMIT for a 1-cm^2 tandem device with an Ag-based screen-printed metallization.

Seite 8 / 11
Page

007072
ISFH CalTeC
2026-07

11 Calibration result - Area

The following calibration result is obtained for the area determination.

Designated illumination area of cell $A_{designated}$	$(3.901 \pm 0.016)\ cm^2$

12 Calibration result - External quantum efficiency

Figure 2 shows the resulting EQE curves of the top (red solid line) and bottom (blue solid line) cell.

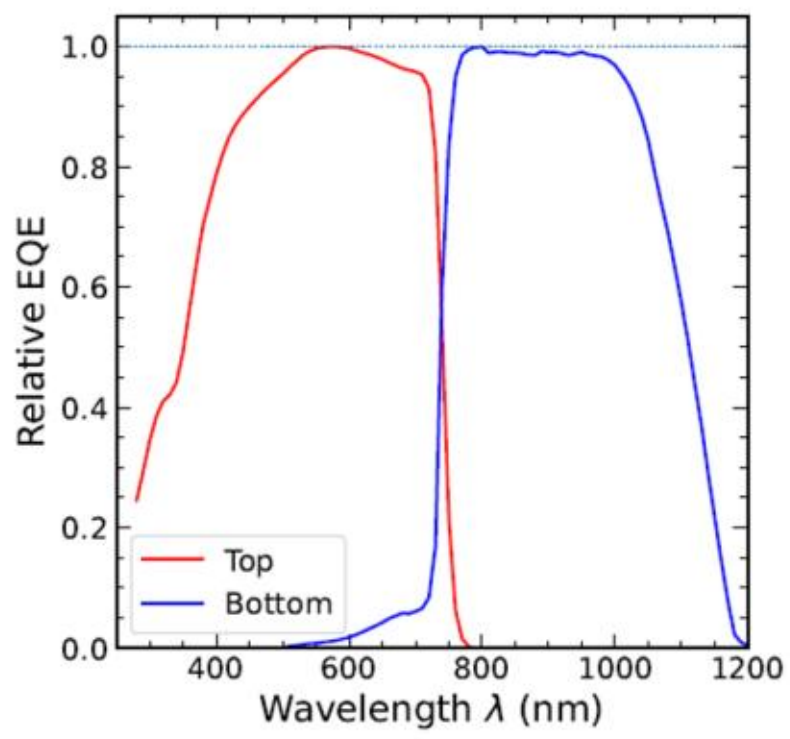

Fig. 2: Plot of the resulting EQE curves of the top (red solid line) and bottom cell (blue solid line).

Seite 9 / 11
Page

007072
ISFH CalTeC
2026-07

13 Calibration results - Maximum power point tracking

The following results are obtained for the maximum power point tracking:

Maximum power P_{final}:	(123.1 ± 2.5) mV
Efficiency η_{final}:	(31.56 ± 0.63) %
Stability value δ:	−0.05 %/min
Stability note:	settled 0.1 %/min

Figure 3 shows the maximum power point tracking over a time window of 600 s.

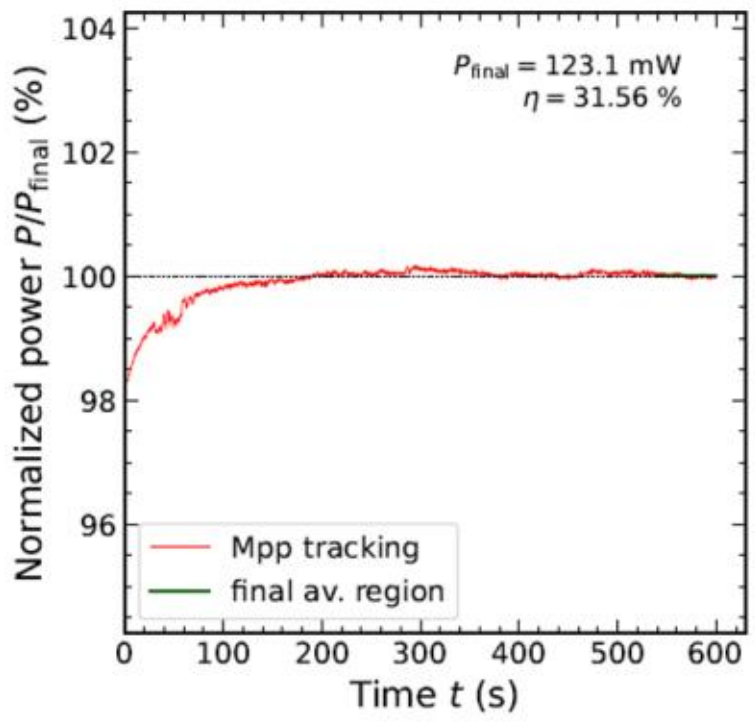

Fig. 3: Plot of the maximum power point tracking over a time window of 600 s.

Seite 10 / 11
Page

007072
ISFH CalTeC
2026-07

14 Current-voltage measurement

The following results are obtained for the characteristic current-voltage parameters as determined from the forward and reverse scan.

Scan direction:	forward scan	reverse scan
Short-circuit current I_{sc}:	(74.3 ± 1.2) mA	(74.4 ± 1.2) mA
Open-circuit voltage V_{oc}:	(1982 ± 12) mV	(1975 ± 12) mV
Fill factor FF:	(81.9 ± 1.3) %	(81.4 ± 1.3) %
Maximum power P_{MPP}:	(120.6 ± 2.4) mW	(119.5 ± 2.4) mW
Efficiency η:	(30.91 ± 0.62) %	(30.63 ± 0.61) %
Hysteresis:	−0.6 %	

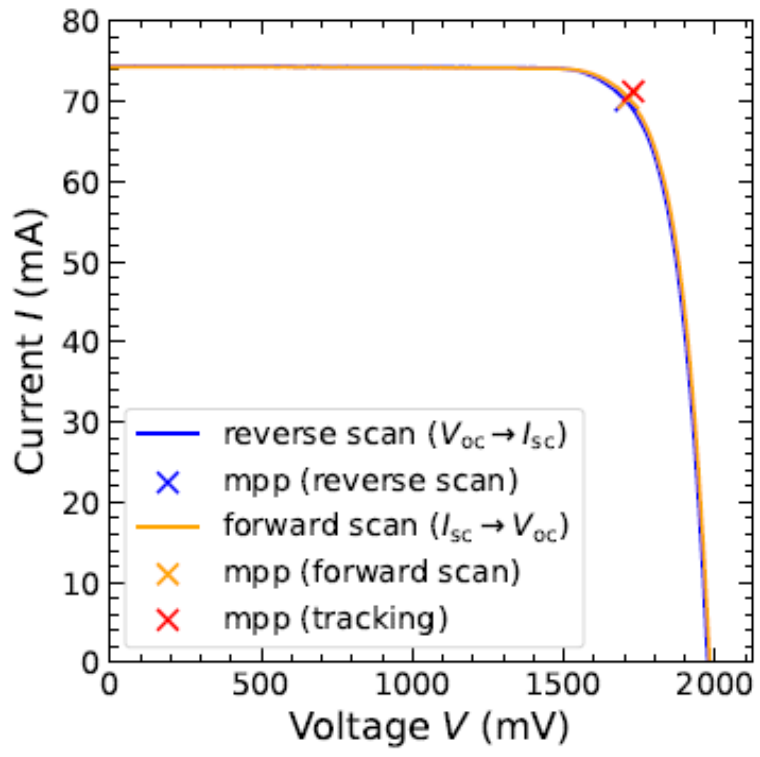

Fig. 4: Plot of the measured current-voltage characteristics under standard test conditions.

Figure S3. Certification results (normalized EQE, MPP, and *JV* data) from ISFH for a 4-cm^2 tandem device with an Ag-based screen-printed metallization.

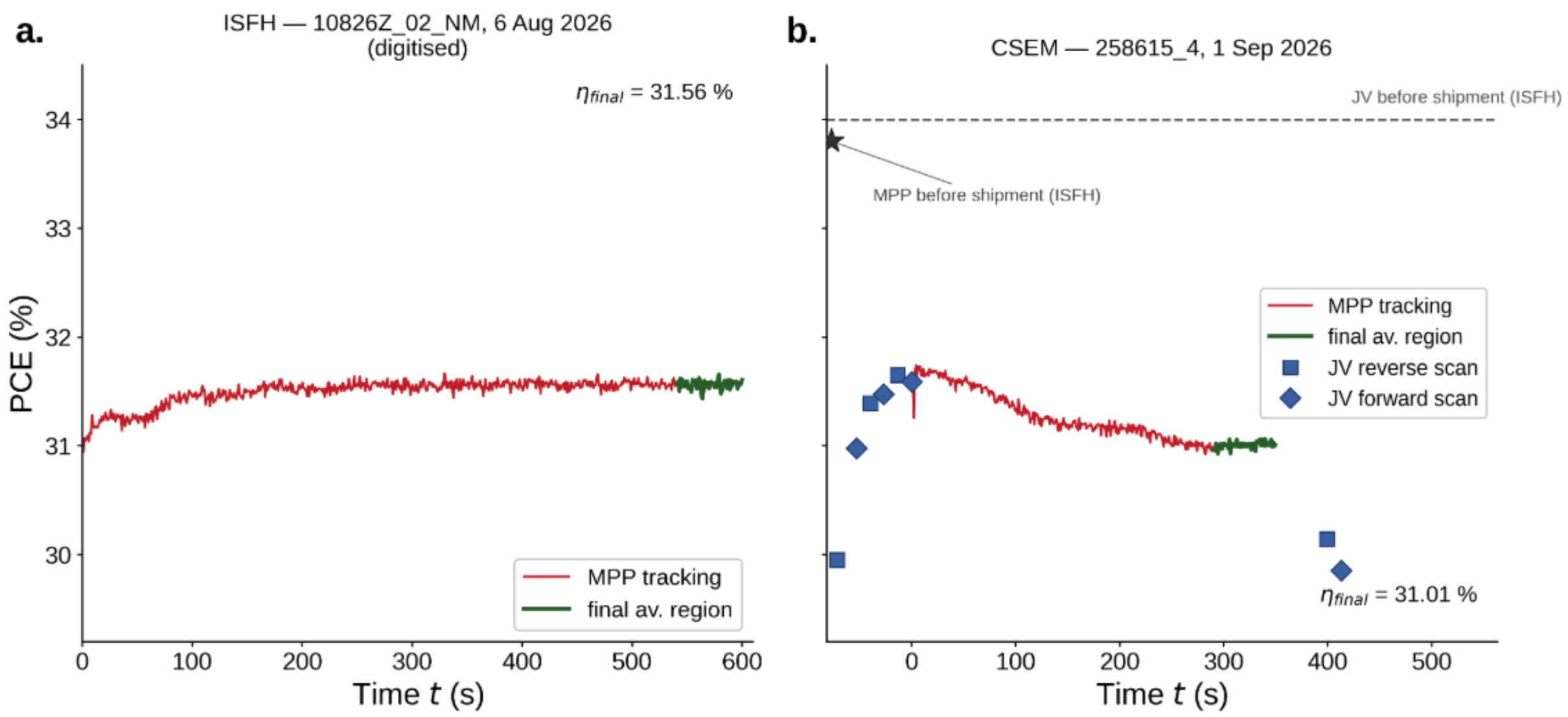

Figure S4. Comparison of *JV* measurements performed at (a) ISFH during certification, (b) CSEM after certification. The results suggest that the cell likely degraded prior to the ISFH measurement. The close agreement between the ISFH measurement and the CSEM post-certification measurement indicates that differences arising from the measurement procedures and spectral mismatch were relatively small.

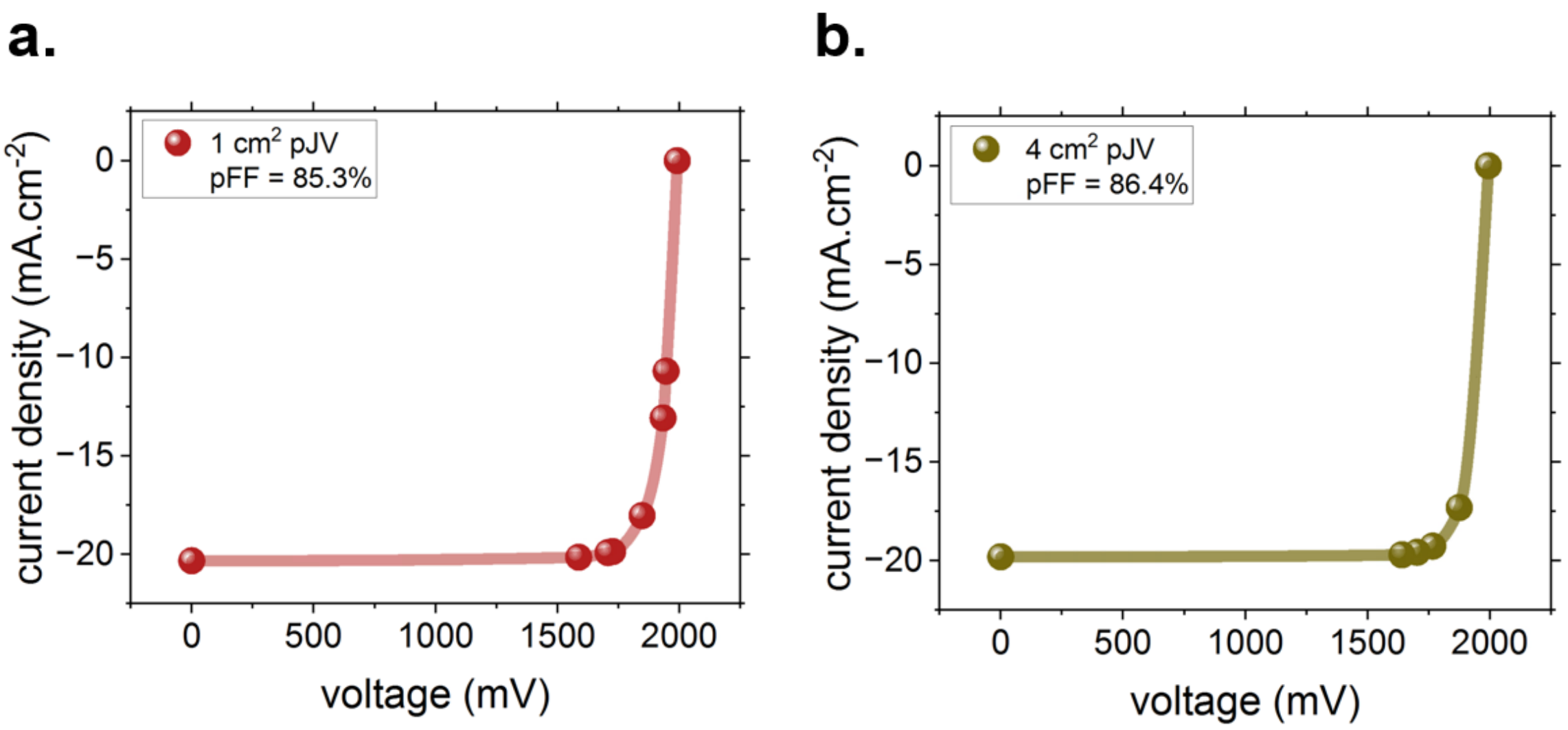

Figure S5. **Pseudo fill factor (pFF) extracted from light-intensity-dependent *JV* measurements.** The 4 cm² device exhibits an approximately 1%$_{abs}$ higher pFF than the 1 cm² device. This improvement originates primarily from an increase in the silicon bottom-cell pFF upon scaling from 1 to 4 cm², while the top-cell contribution remains essentially unchanged. The enhanced tandem pFF indicates that the top-cell quality is preserved during scaling and does not suffer from degradation associated with increased shunt losses.

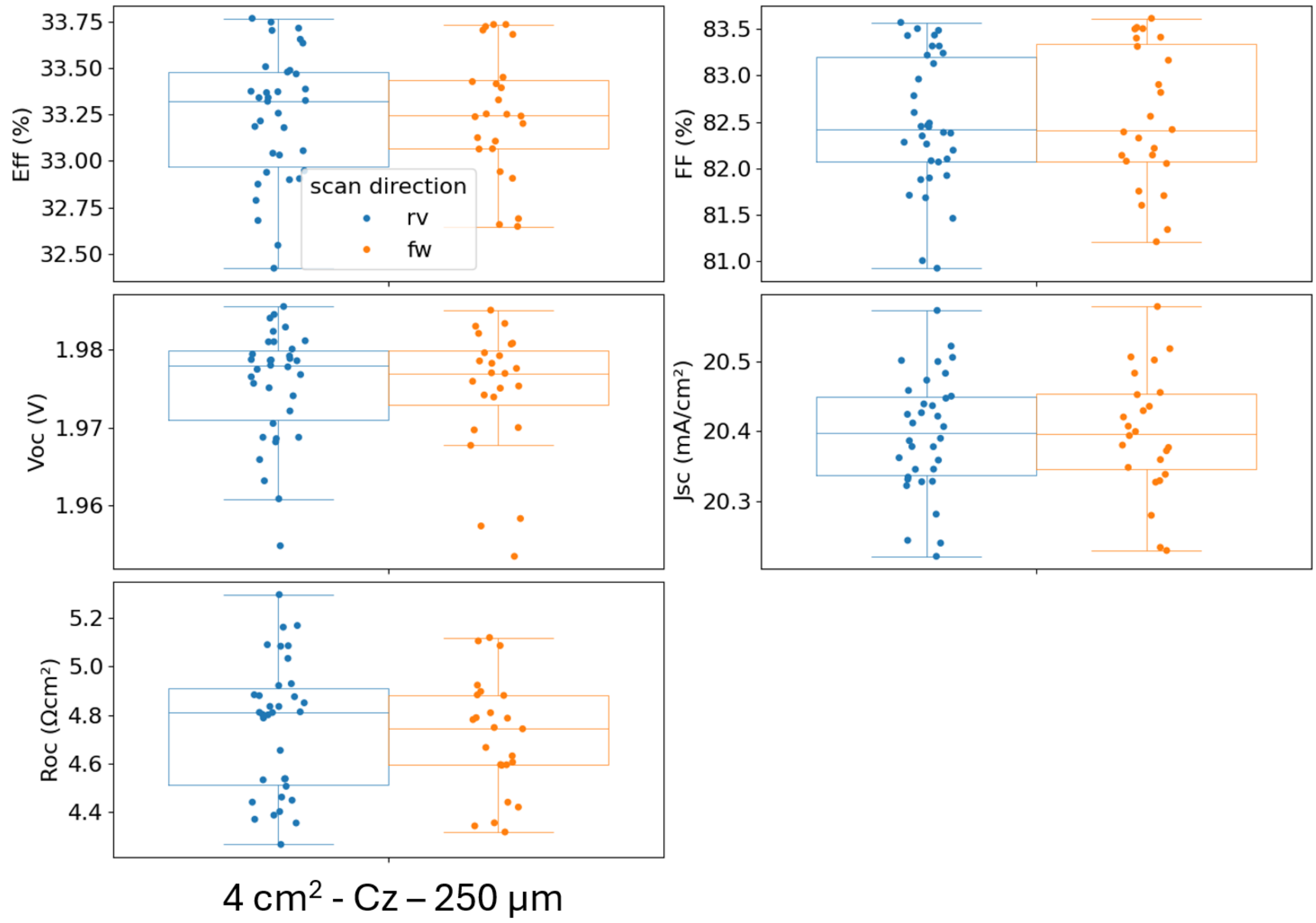

Figure S6. 4 cm^2 tandems featuring thick Cz wafers using an Ag screen-printed metallization.

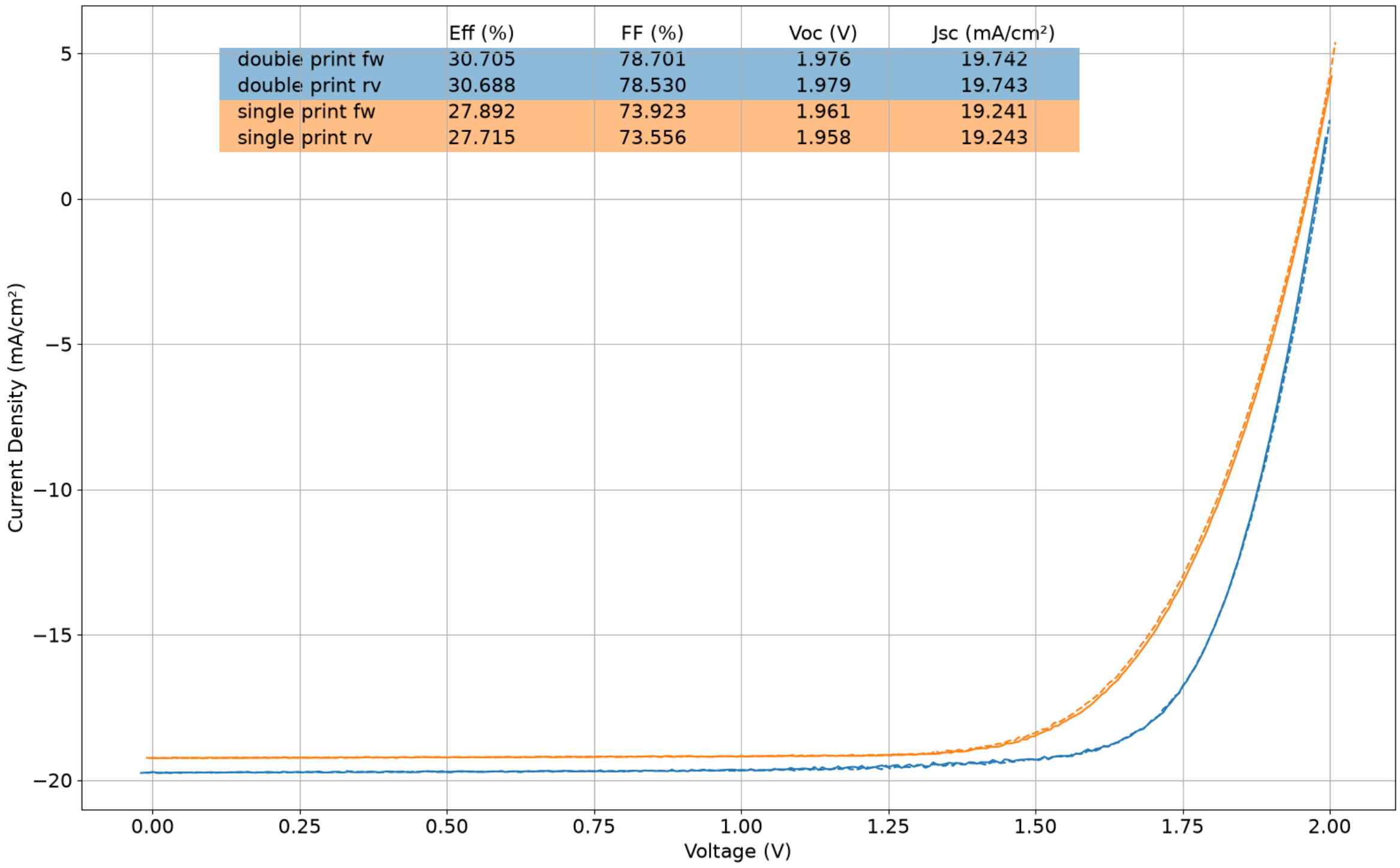

Figure S7. Comparison of the performance of 54 cm^2 tandem cells with either single or double print: the FF significantly increases after double printing due to reduced series resistance losses in the metallization grid, most likely due to reduced finger line resistance. Note that shadowing induced by the pogo-bars is not corrected here.

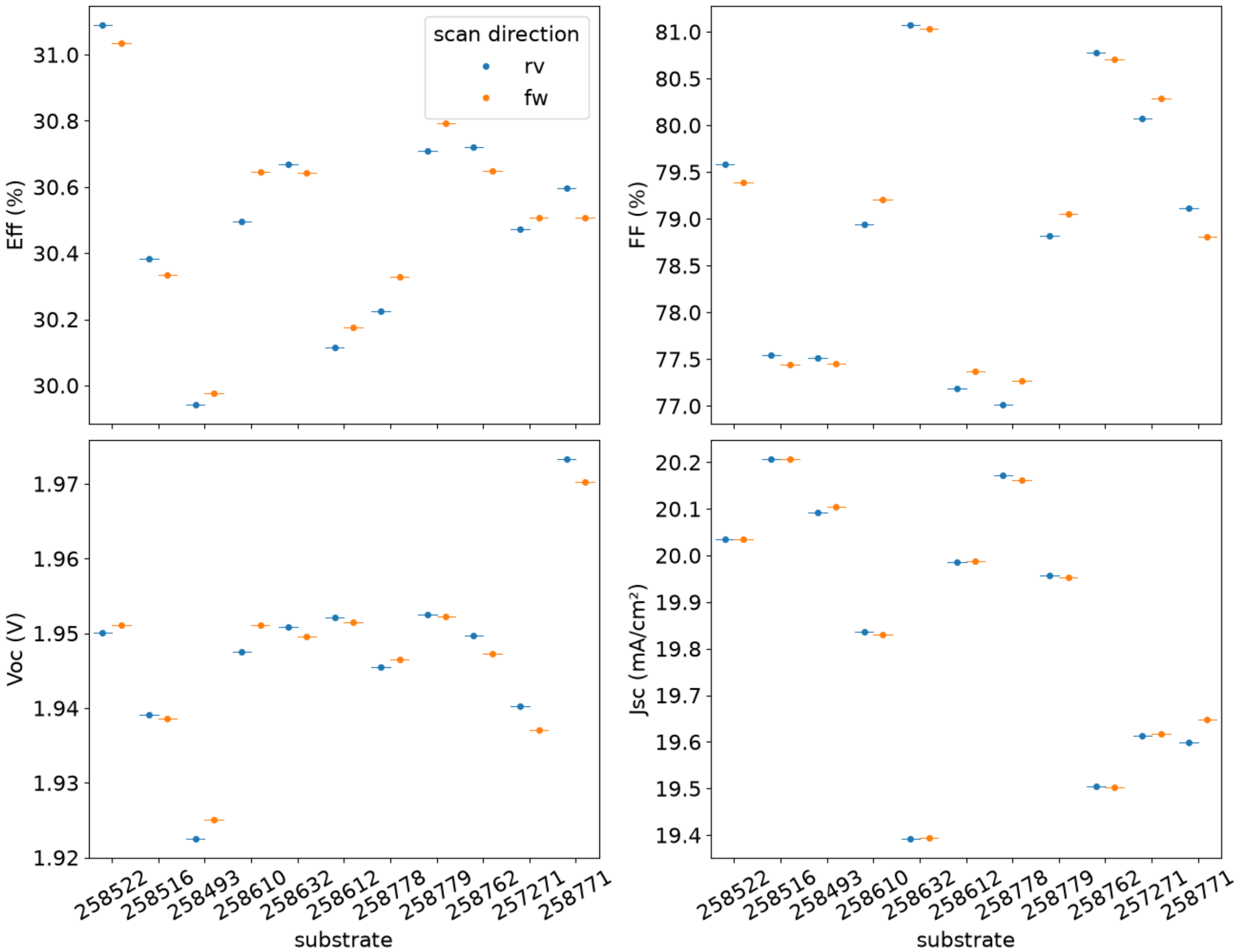

Figure S8. Performance statistics of eleven 54 cm² devices fabricated using a double-print metallization process. Note that shadowing induced by the pogo-bars is not corrected here.

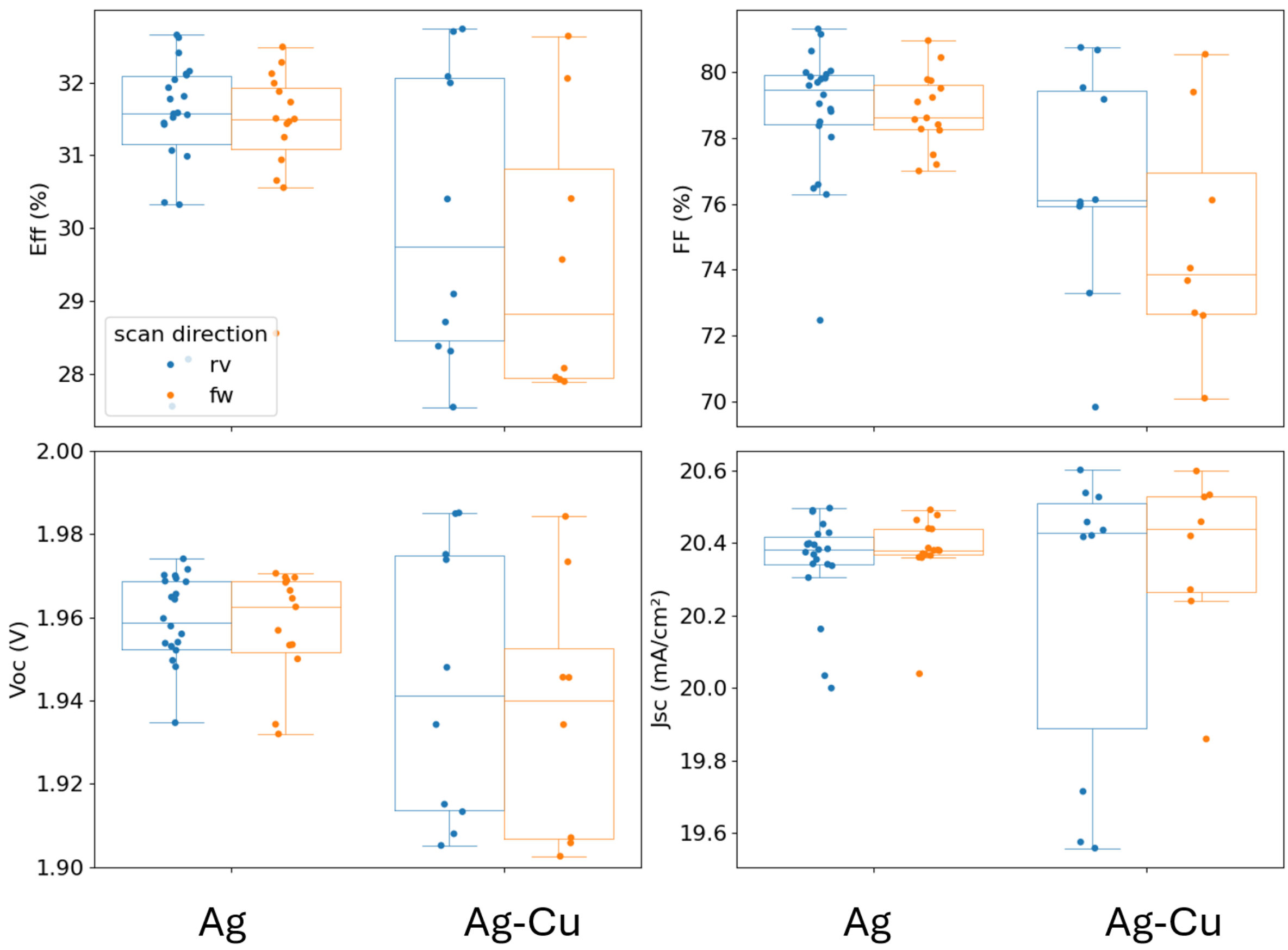

Figure S9. Device performance of 1-cm^2 tandems with either Ag or Ag-Cu screen-printed metallization.

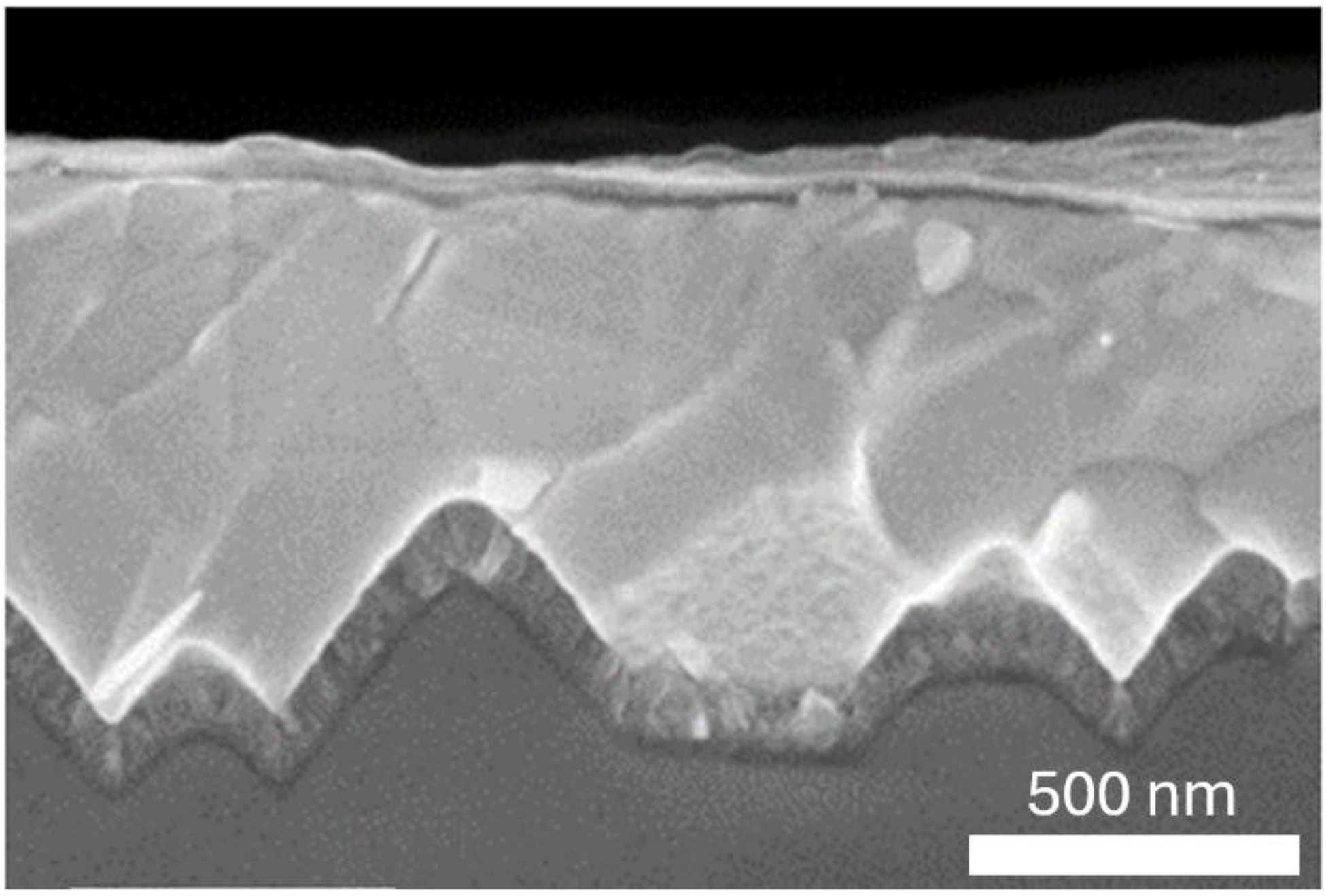

Figure S10. Cross section SEM of a nanotextured perovskite-silicon tandem highlighting how the non-conformal perovskite coating effectively planarizes the textured silicon surface.

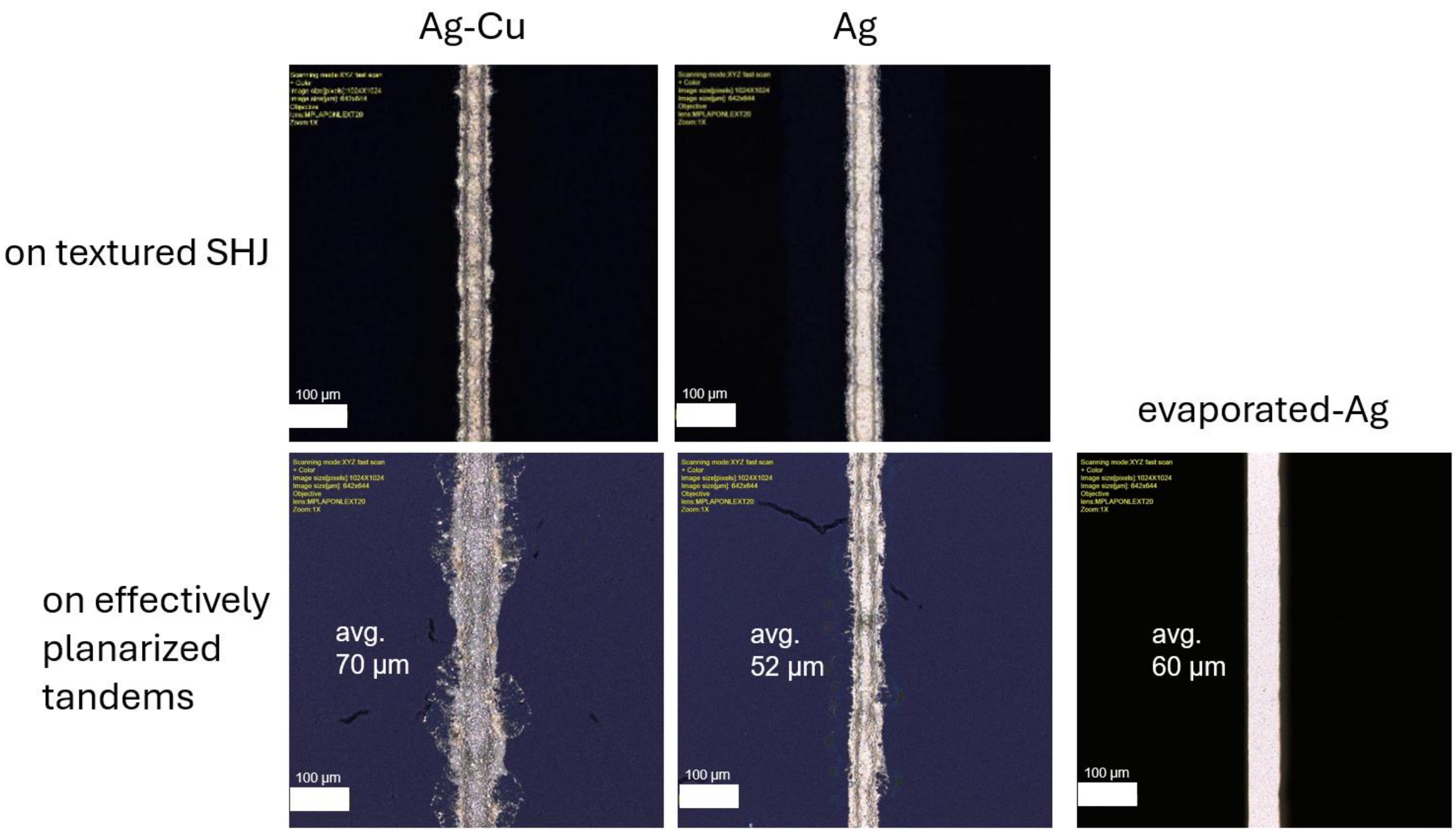

Figure S11. Comparison of finger spreading for screen-printed Ag and Ag–Cu metallization on textured SHJ cells and front-side planarized tandem cells, in comparison with evaporated Ag contacts. The Ag paste exhibits similar spreading behaviour on planarized and textured surfaces, whereas the Ag–Cu paste shows greater spreading on the planar tandem front side. ITO is used as the front TCO for all the tandem samples presented here.

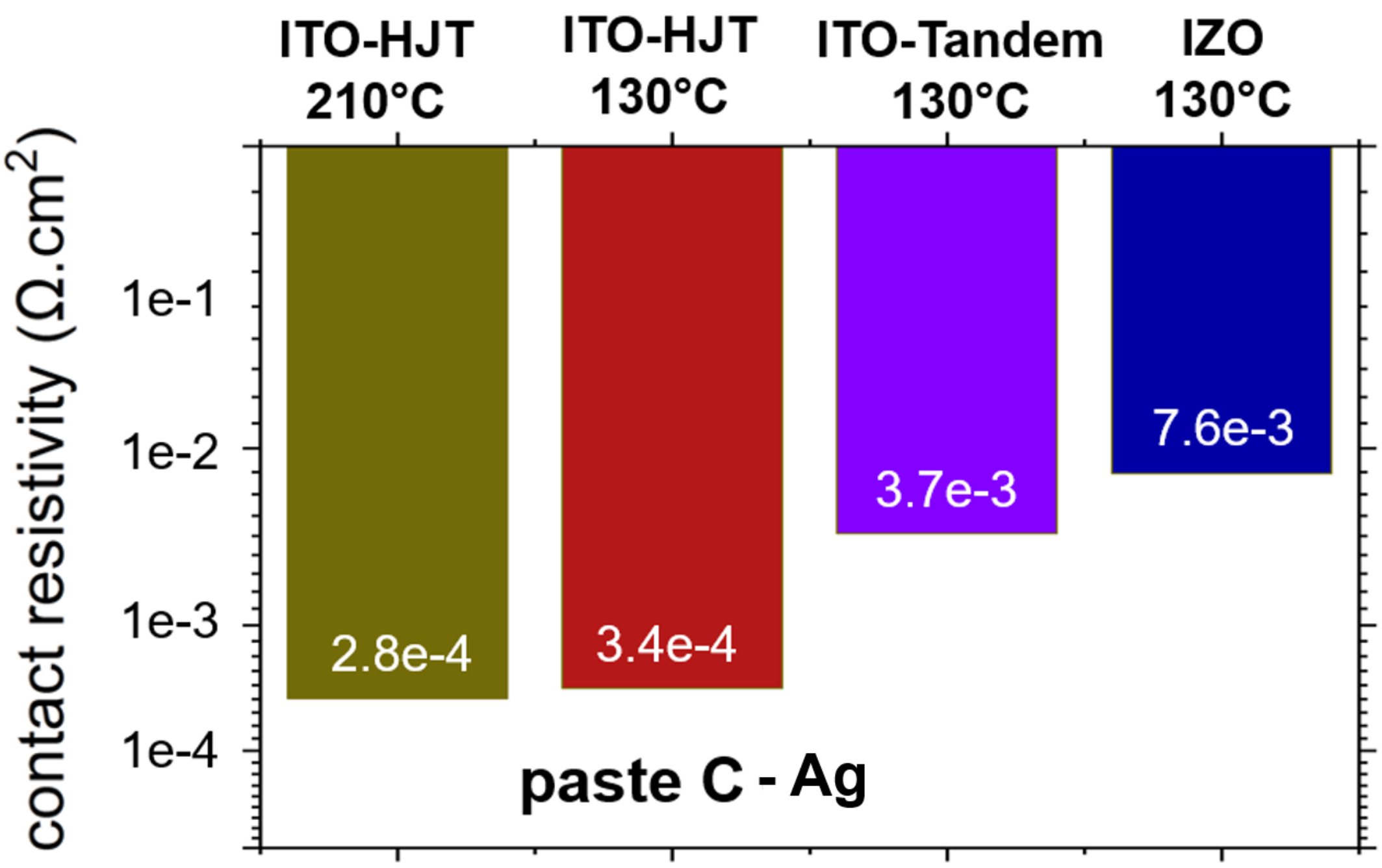

Figure S12. Comparison of the contact resistivity of an Ag paste on thick silicon heterojunction technology (HJT)-grade ITO, thin tandem-grade ITO (40 nm), and IZO, as a function of curing temperature.

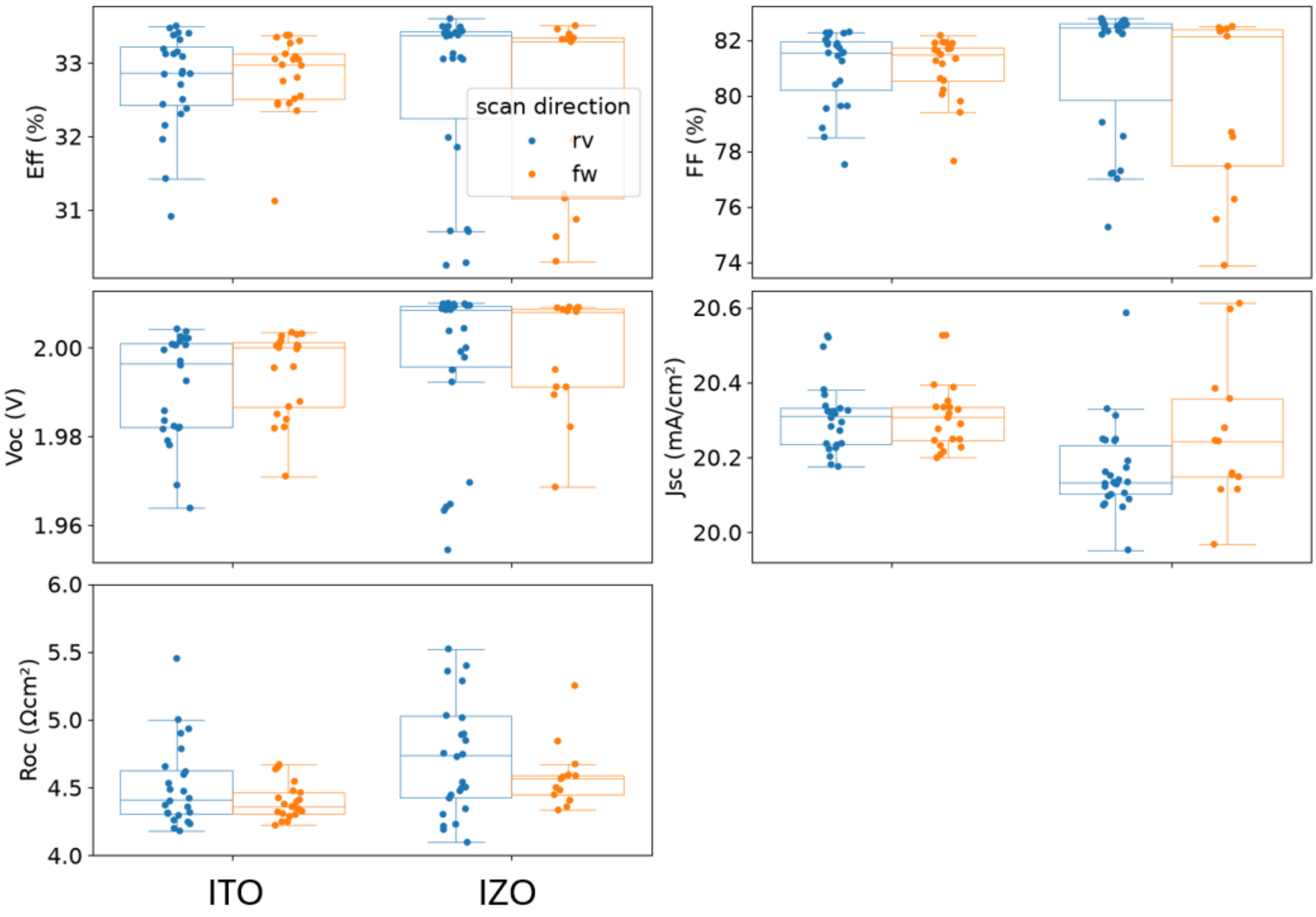

Figure S13. Device performance (1 cm^2 on 4-inch wafers) with ITO and IZO, showing that R_{oc} slightly increases likely due to increased contact resistivity at the metal-IZO interface.

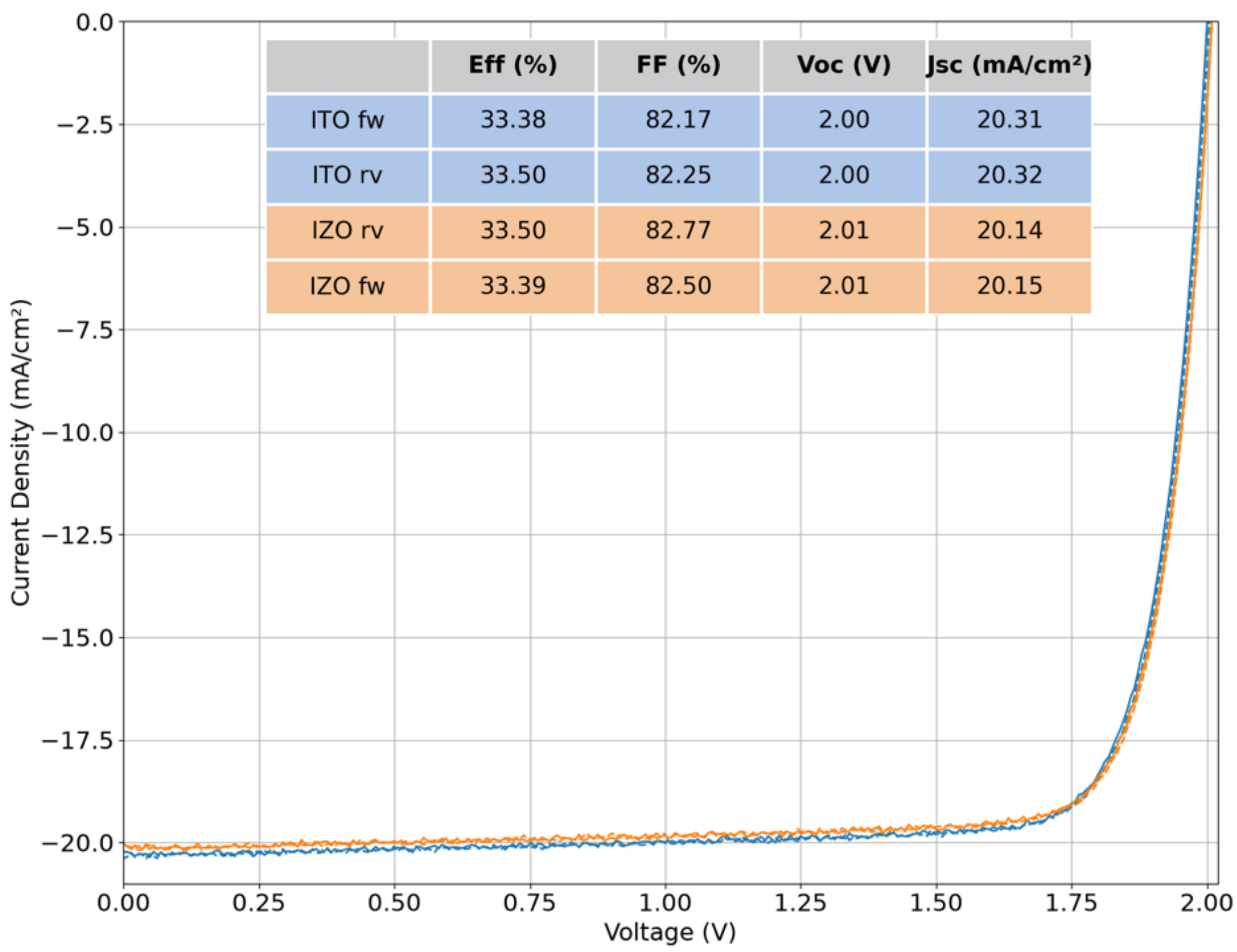

Figure S14. Champion forward- and reverse-scan JV curves for 1 cm^2 tandem devices employing ITO or IZO.

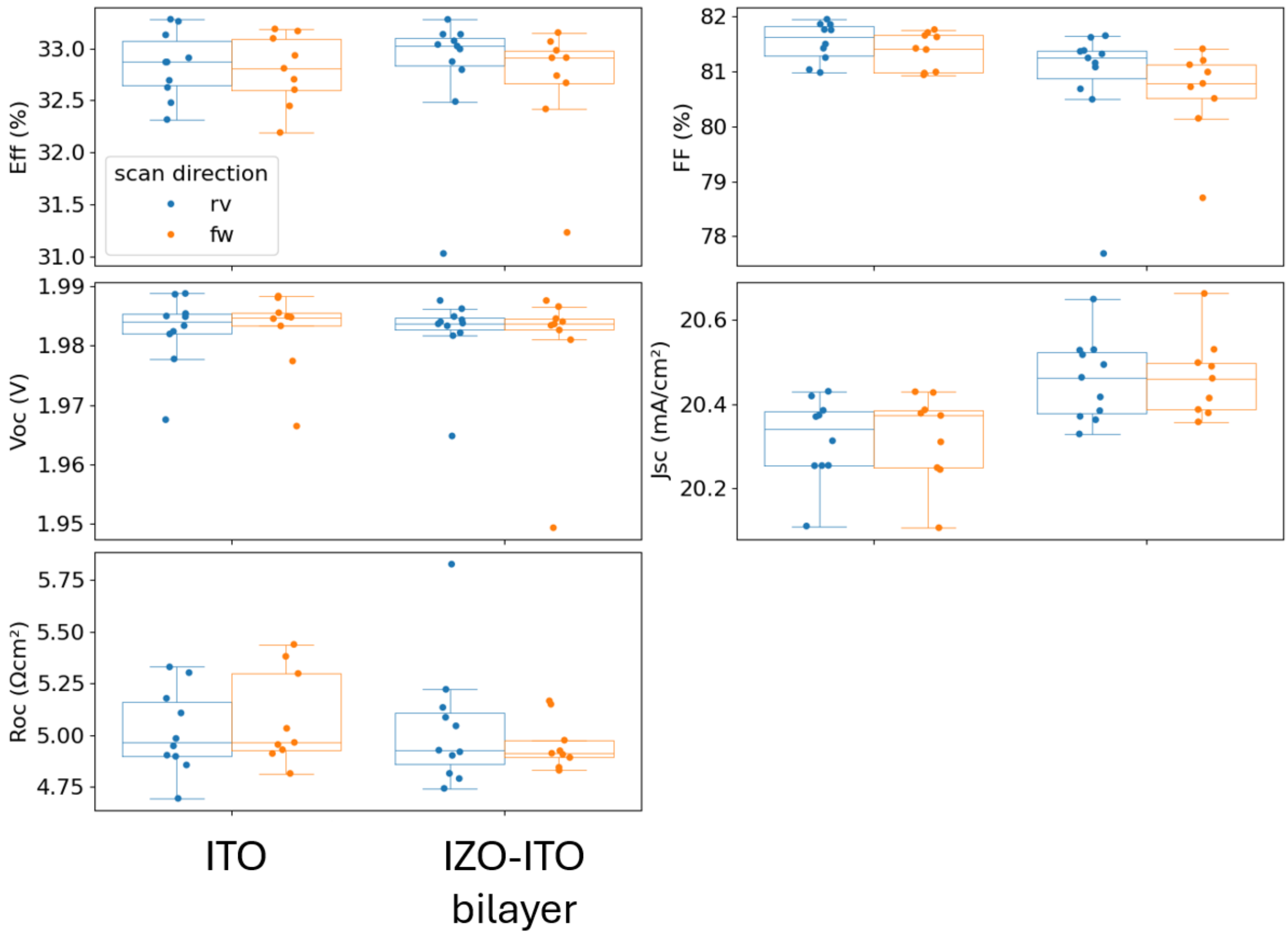

Figure S15. Device performance (1 cm^2 on 4-inch wafers) when using ITO or an IZO-ITO bilayer. With the bilayer, 20 nm of IZO is capped with 20 nm of ITO to improve the contact resistance with the Ag paste.

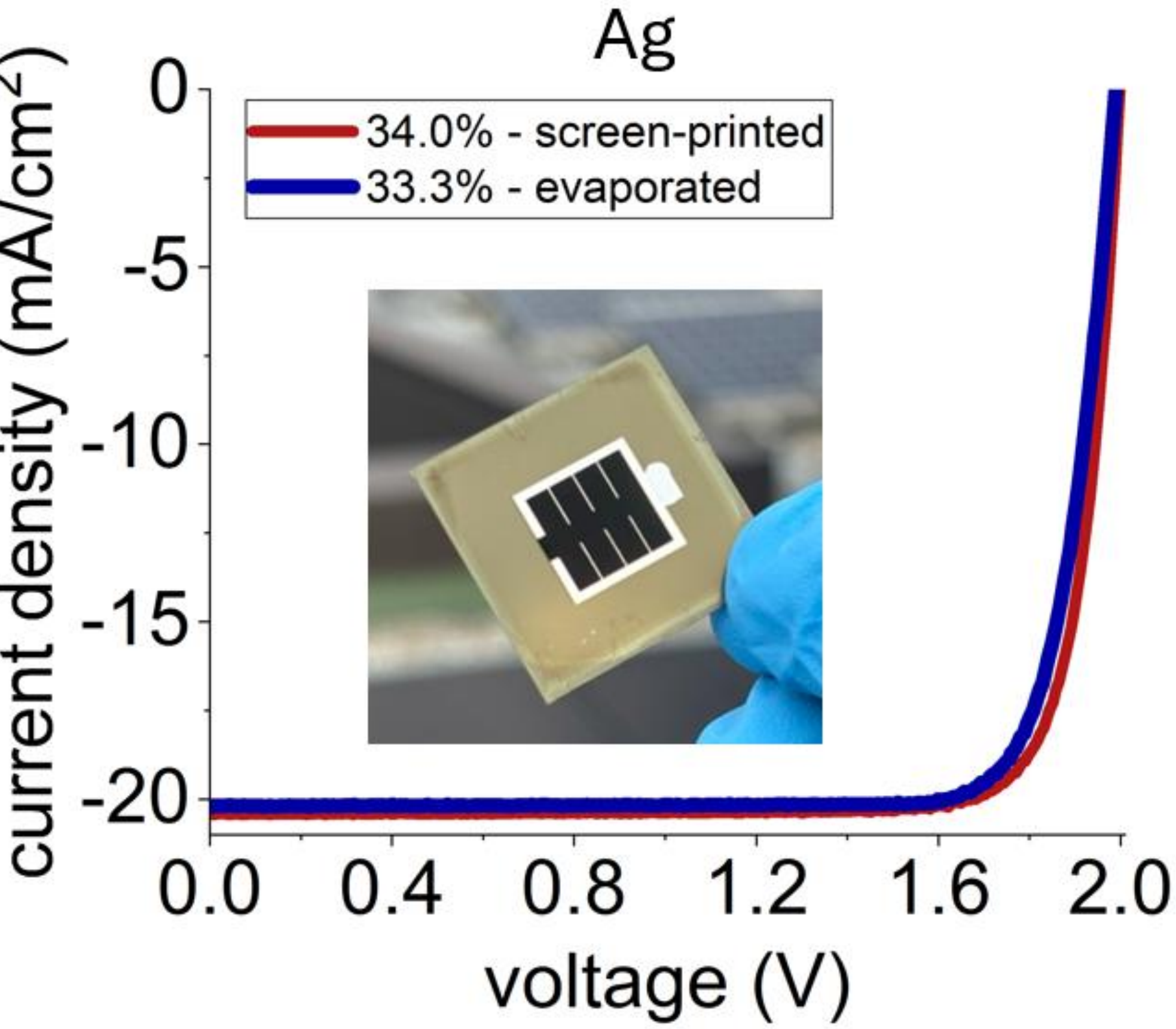

Figure S16. Champion reverse-scan *JV* curves of 1 cm² devices featuring screen-printed or evaporated Ag electrodes. An image of the evaporated Ag metallization is shown in the inset.

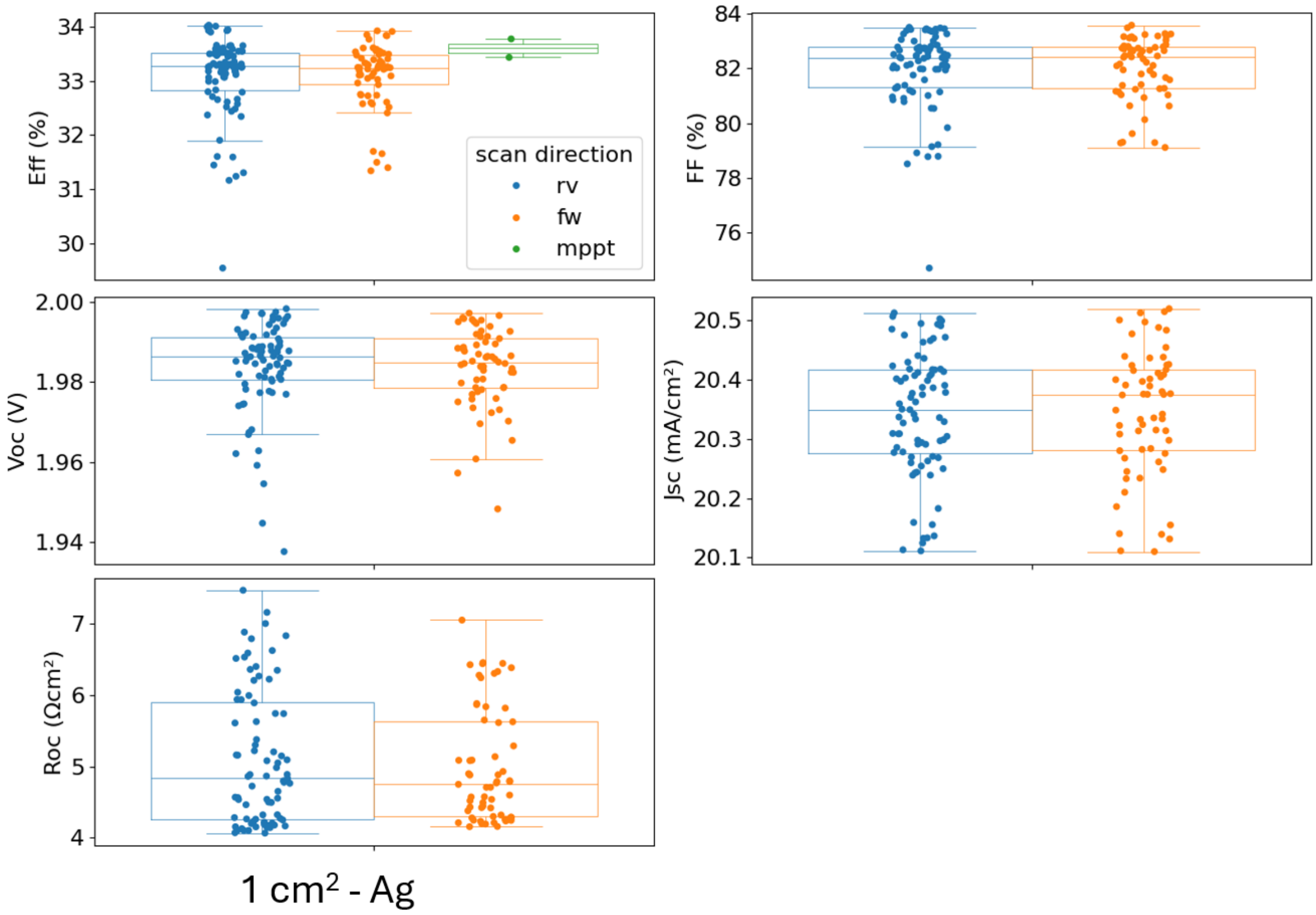

Figure S17. Statistical distribution of the performance parameters of 1 cm^2 tandem cells fabricated with an Ag screen-printed metallization.

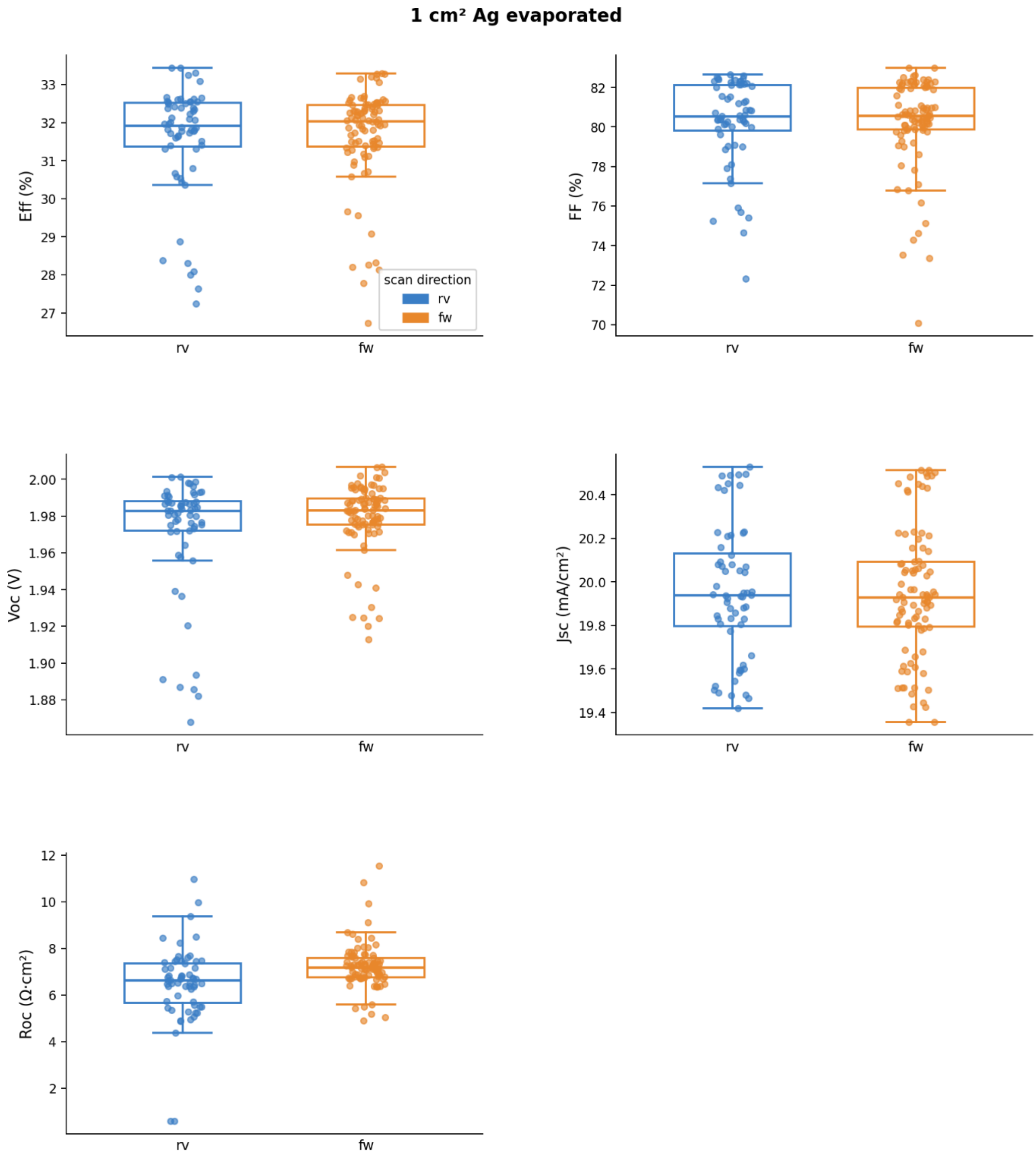

Figure S18. Statistical distribution of the performance parameters of 1 cm^2 tandem cells fabricated with an evaporated Ag grid.

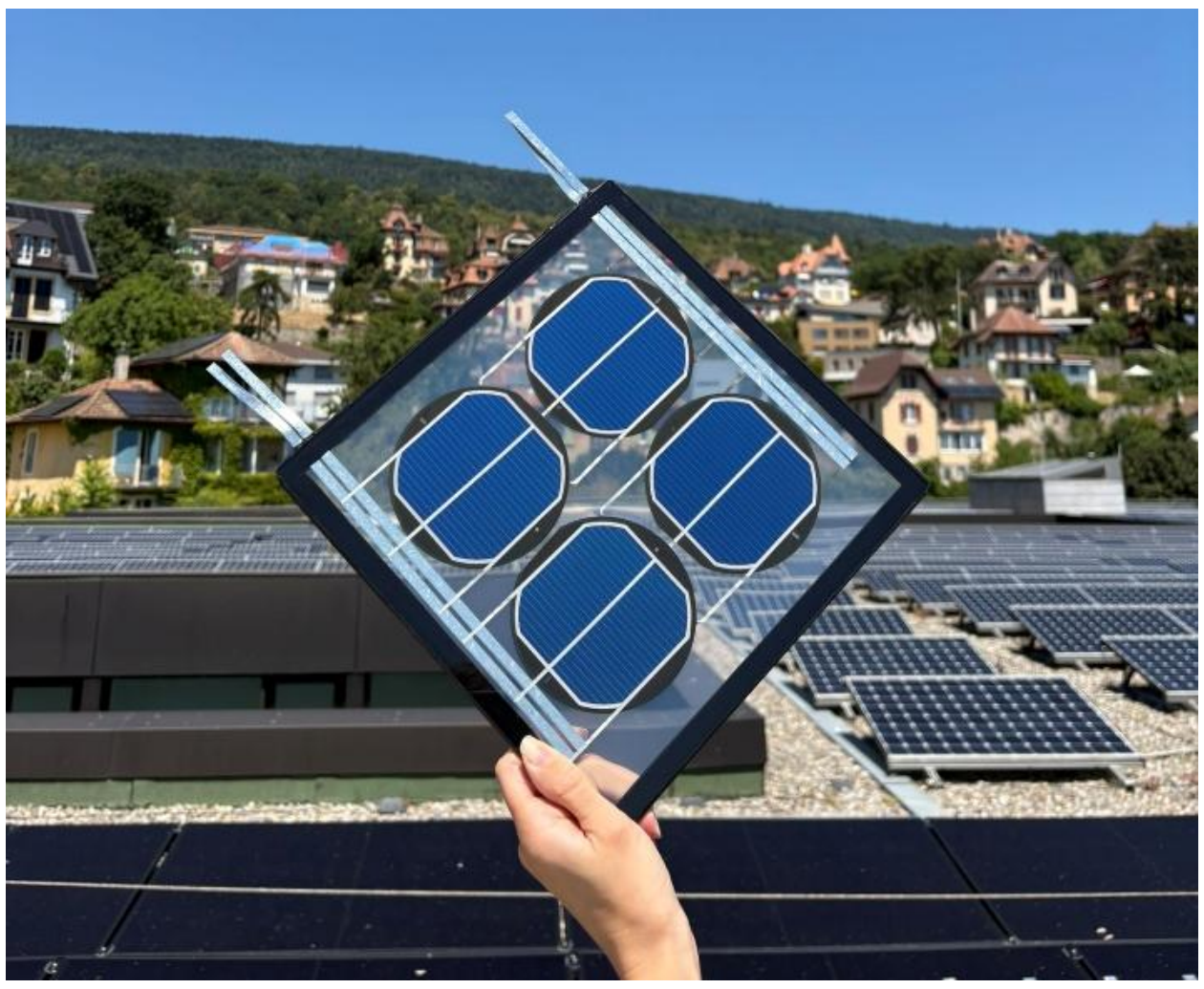

Figure S19. Photograph of the 4-cell module with a total active area of 216 cm^2.

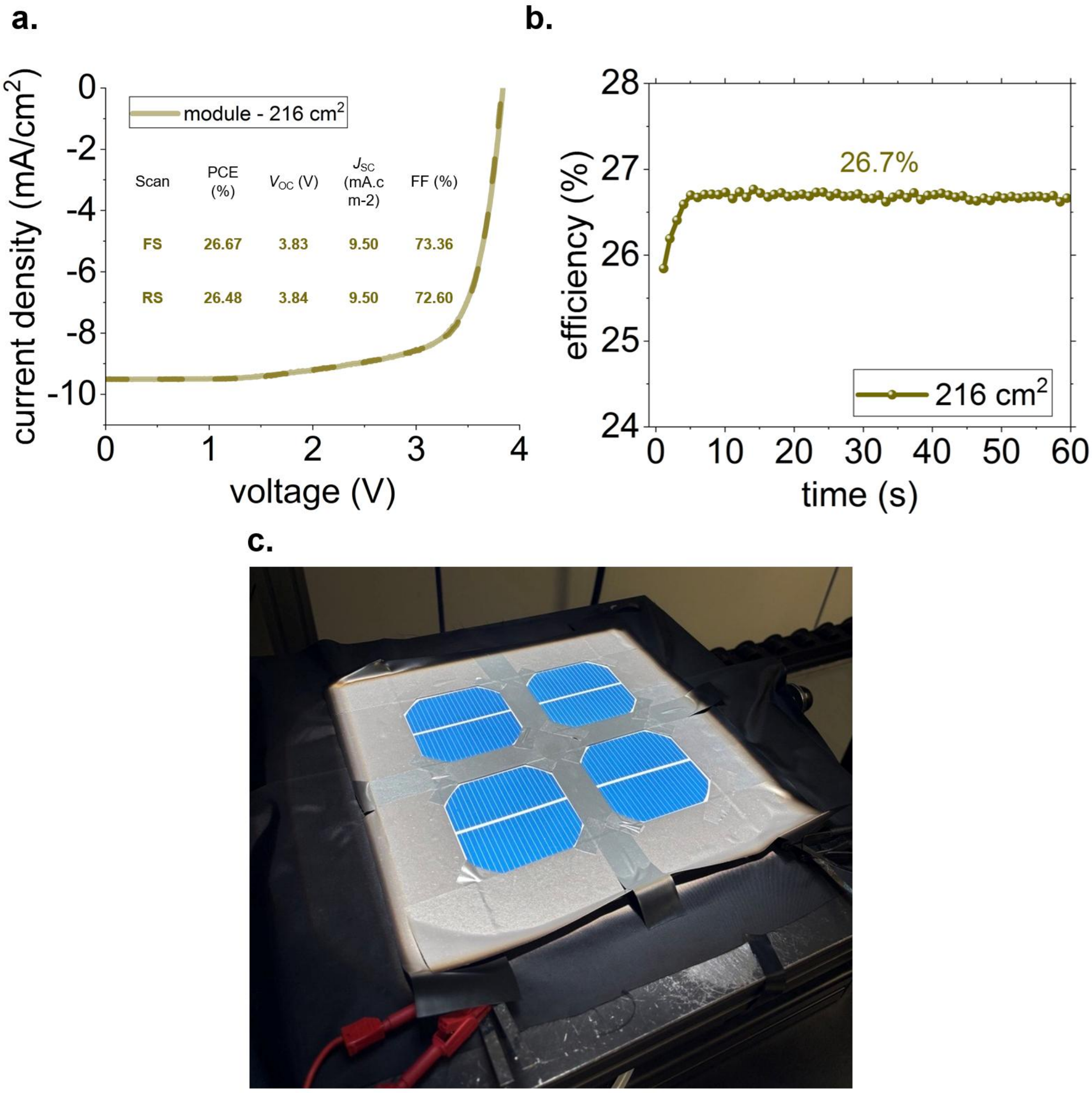

Figure S20. Performance and photograph of an encapsulated 216-cm² tandem mini-module. **(a)** Reverse and forward *JV* data under standard AM1.5G illumination **(b)** Stabilized MPP tracking, yielding an efficiency of 26.7% (designated illumination area). **(c)** Photograph of the module consisting of four 54 cm² tandem cells interconnected in two parallel strings of two cells, resulting in a total active area of 216 cm². Masking was used to record *JV* properties.

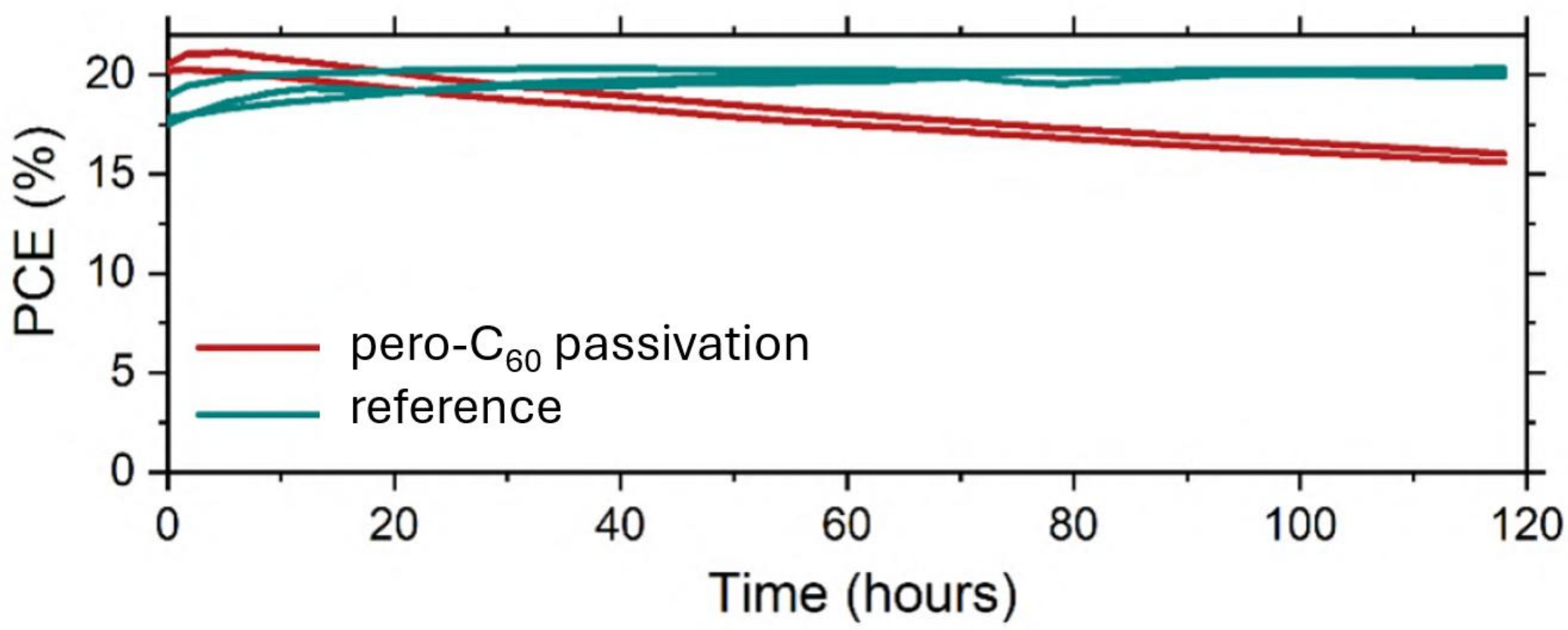

Figure S21. Indoor accelerated aging (at 35°C in N_2) of unencapsulated perovskite single-junction cells with 1.65 eV tandem-relevant absorber, with and without perovskite-C_{60} interface passivation. Passivation, despite initial PCE gain, accelerates degradation. To quantify the influence of metal paste on stability, we simplified the device stack and removed the passivation interlayer.

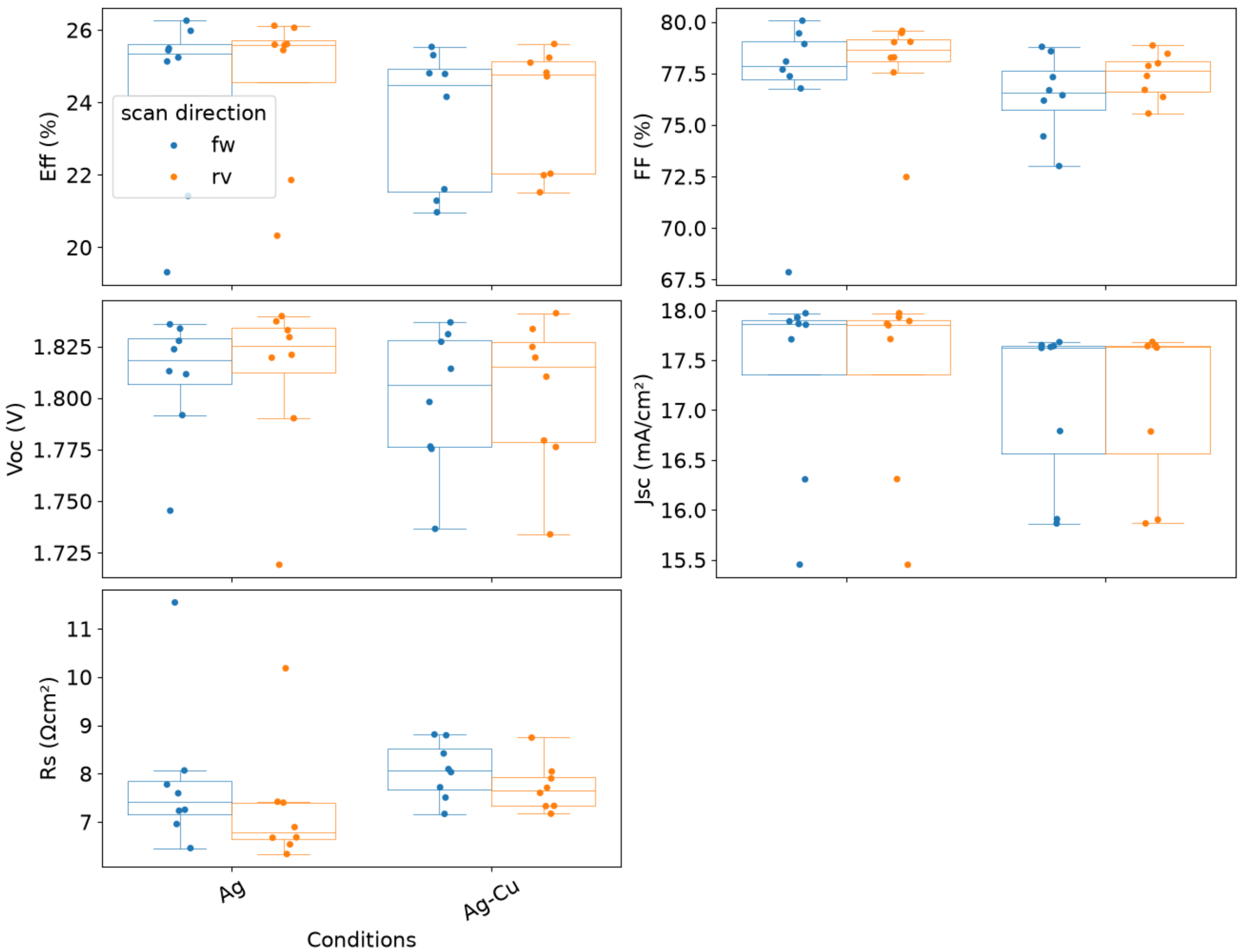

Figure S22. Device performance statistics of encapsulated 1 cm^2 cells with the stability stack (no interface passivation) measured before the start of the light-soaking experiment in standard test conditions. Due to the absence of perovskite/C60 passivation, the achieved V_{OC}s are limited to 1.83V.

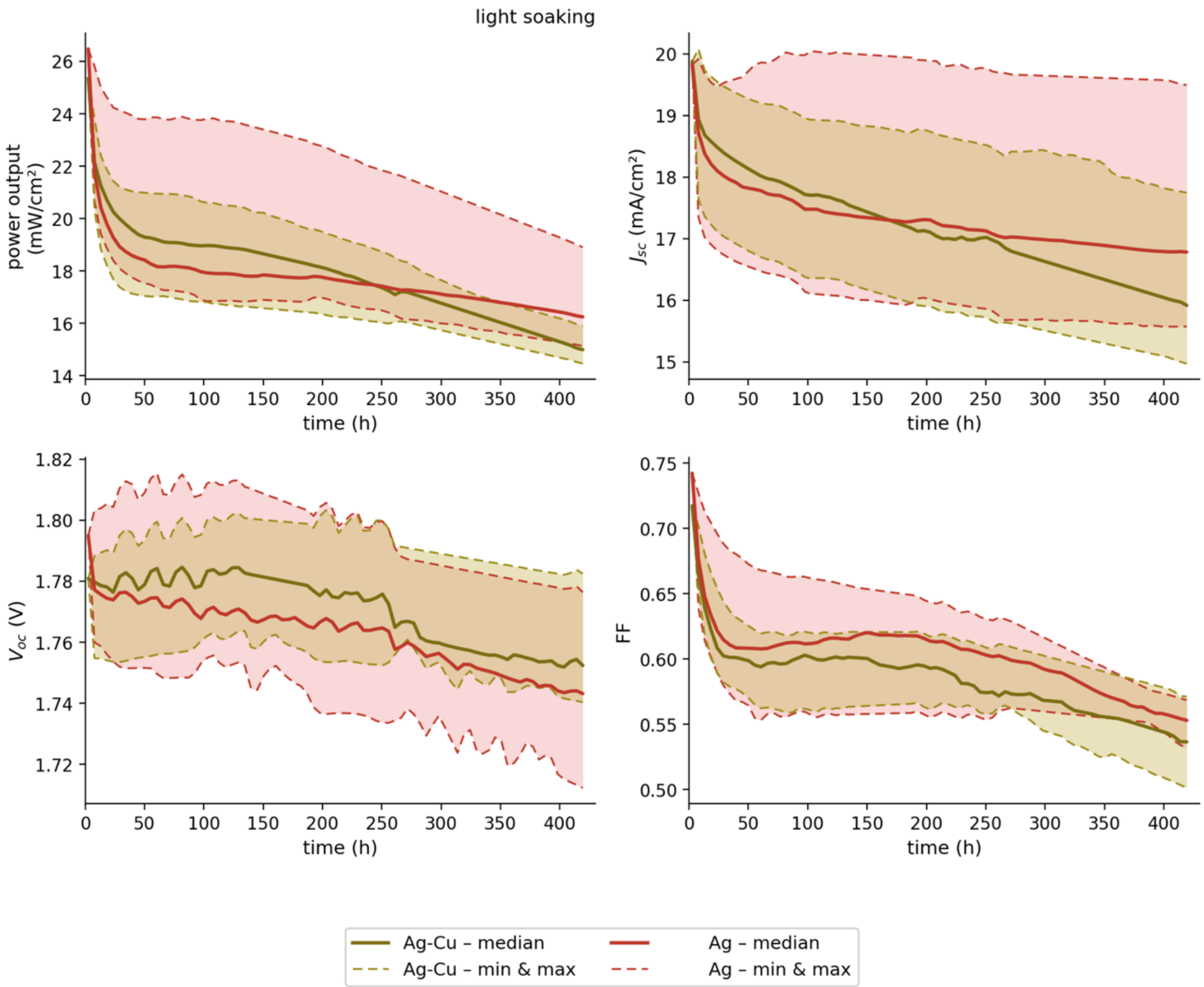

Figure S23. Evolution of the *JV* performance parameters during light soaking at V_{oc} and 55 °C for 1 cm^2 tandems with Ag or Ag-Cu paste. Full *JV* curves were acquired every 15 min.

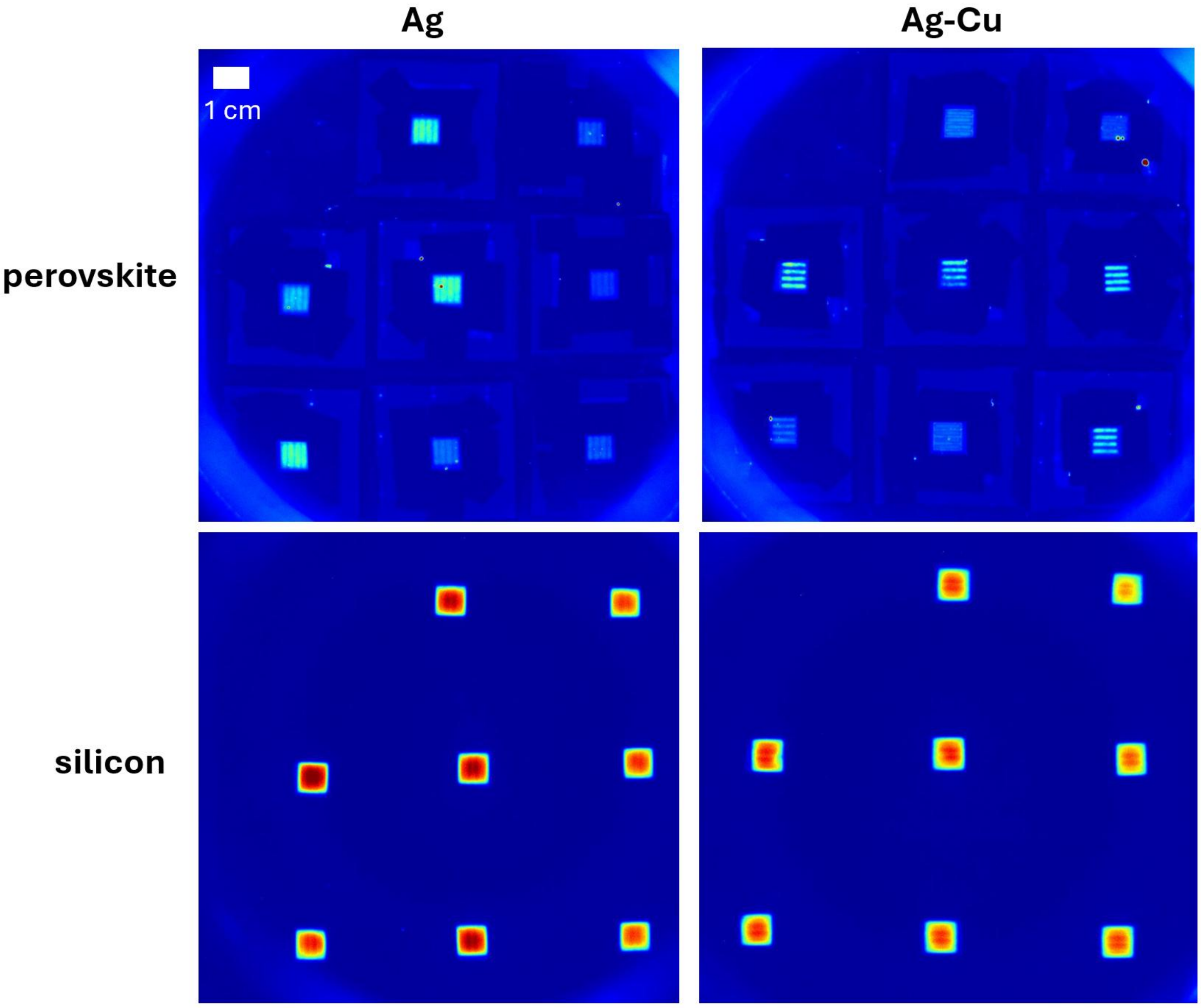

Figure S24. Photoluminescence (PL) images of the perovskite top and silicon bottom cells after light soaking (samples from Fig.2a), where Ag samples show slightly higher PL response.

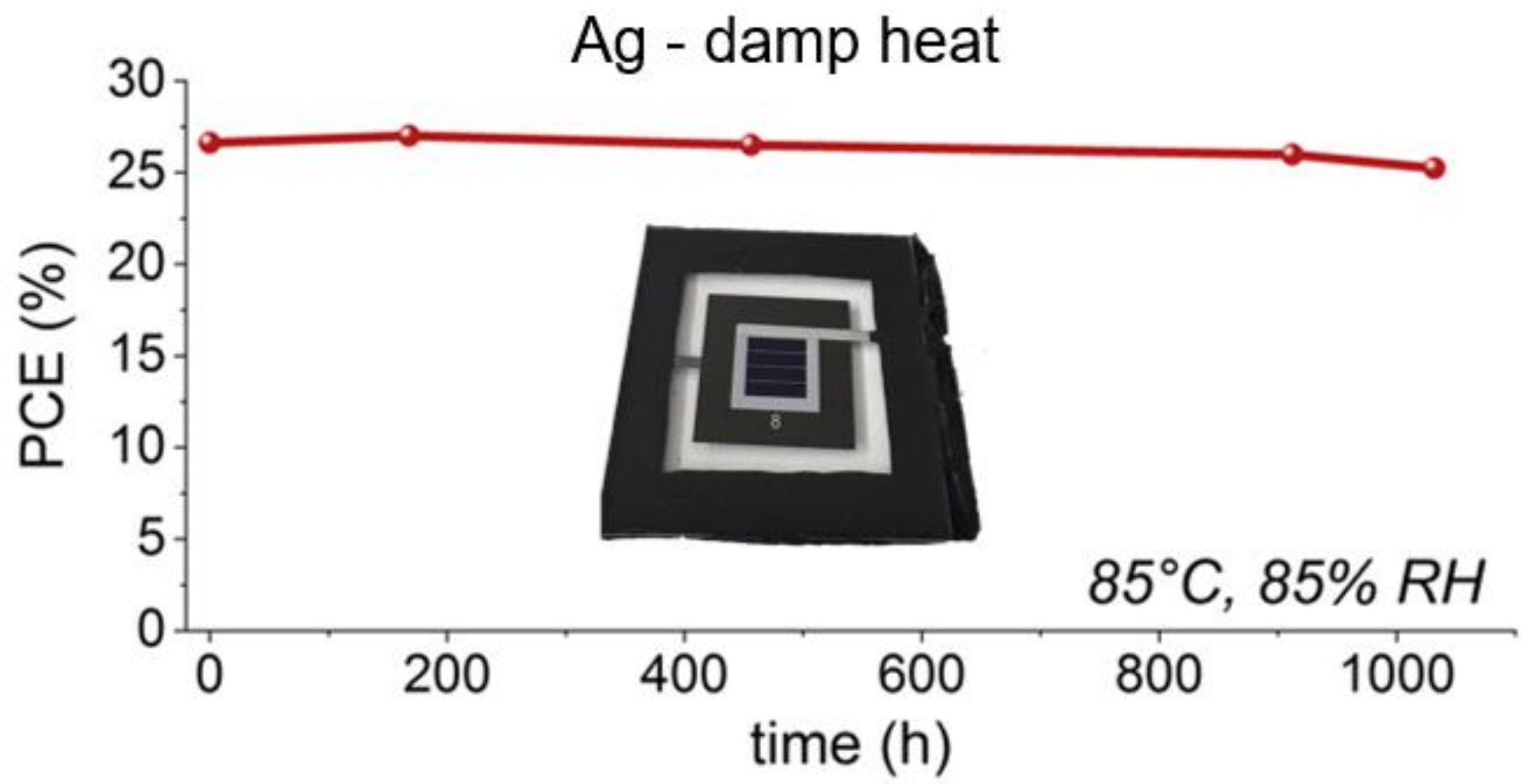

Figure S25. **Damp-heat stability testing.** Encapsulated 1-cm^2 tandem solar cells employing an Ag screen-printed metallization were subjected to damp-heat aging at 85 °C in 85% relative humidity for 1000 h. Device performance was periodically monitored through *JV* measurements.

Table S1. Properties of the TCO films employed in this work. As-deposited values are reported here. The O_2 concentration differs between ITO and ITO-optimized.

	mobility ($cm^2.V^{-1}.s^{-1}$)	carrier density (cm^{-3})	R_{sheet} (ohm/sqr)	thickness (nm)
**ITO**	41	2.97e20	138	39
**ITO-optimized**	56	1.94e20	167	36
**IZO**	46	2.79e20	144	35

Table S2. Details of different device stacks utilized in this work

Parameter	High-efficiency stack	Stability stack	TLM stack
Purpose	Record efficiency demonstration	Reliability assessment (TC, DH, light soaking)	Contact resistance extraction
Perovskite-C_{60} passivation	Yes	No	No
SnO_x thickness	17-20 nm	35 nm	-
ARC	Yes, after TCO	No	No
Encapsulation	No	Yes	No
Metallization	Ag / Ag-Cu	Ag / Ag-Cu	Ag / Ag-Cu
Front metallization	Screen printed	Screen printed	Screen printed
Rear contact	Sputtered Ag	Sputtered Ag	-
Back reflector	Yes	No	No
Device area	1-4-54 cm^2	1 cm^2	TLM geometry
Typical PCE before or after encapsulation	31 to 34% before encapsulation	~26% after encapsulation	N/A

References

1. M. Boccard, L. Antognini, J. Cattin, J. Dreon, W. Lin, V. Paratte, D. Turkay, C. Ballif, Loss Analysis of a 24.4%-Efficient Front-Junction Silicon Heterojunction Solar Cell and Opportunity for Localized Contacts. *IEEE J. Photovolt.* **13**, 663–671 (2023).

2. A. Descoeudres, Z. C. Holman, L. Barraud, S. Morel, S. De Wolf, C. Ballif, >21% Efficient Silicon Heterojunction Solar Cells on N-and P-Type Wafers Compared. *IEEE J. Photovolt.* **3**, 83–89 (2013).

3. D. Turkay, N. Blondiaux, M. Boccard, K. Artuk, D. A. Jacobs, J. Gay, Q. Jeangros, C. Ballif, C. M. Wolff, Self-Aligned Silica Nanoparticle Rear Reflectors for Single-Junction Si and Perovskite-Si Tandem Solar Cells. *Solar RRL*, doi: 10.1002/solr.202400704 (2025).

4. D. Turkay, K. Artuk, X. Y. Chin, D. A. Jacobs, S. J. Moon, A. Walter, M. Mensi, G. Andreatta, N. Blondiaux, H. Lai, F. Fu, M. Boccard, Q. Jeangros, C. M. Wolff, C. Ballif, Synergetic substrate and additive engineering for over 30%-efficient perovskite-Si tandem solar cells. *Joule* **8**, 1735–1753 (2024).

5. K. Artuk, A. Oranskaia, D. Turkay, F. Saenz, M. D. Mensi, M. De Bastiani, A. F. Castro-Méndez, J. Hurni, C. Allebé, M. Othman, L. Champault, A. G. Kuba, A. Levtchenko, D. A. Jacobs, J. B. Puel, D. Ory, F. Lang, A. Hessler-Wyser, U. Schwingenschlogl, Q. Jeangros, C. Ballif, C. M. Wolff, 60 cm2 perovskite-silicon tandem solar cells with an efficiency of 28.9% by homogeneous passivation. *Nature Communications* **16** (2025).

6. K. Artuk, D. Turkay, A. Kuba, S. Riemelmoser, J. A. Steele, J. Hurni, J. Spitznagel, H. Quest, M. De Bastiani, J. Zhao, J. Diekmann, C. Ongaro, M. Othman, M. Heydarian, O. Fischer, H. Lai, J. S. Austin, S. Zeiske, R. López-Arteaga, C. Liu, M. D. Mensi, A.-F. Castro-Méndez, M. Li, T. W. Gries, S. Hill, F. Saenz, L. Champault, H. A. Can, M. R. Golobostanfard, U. Desai, P. Remondeau, E. Solano, G. Portale, A. Faes, F. Lang, A. Musiienko, N. Rolston, F. Fu, M. C. Schubert, F. Schindler, B. Chen, A. Pasquarello, E. H. Sargent, A. Hessler-Wyser, Q. Jeangros, C. Ballif, C. M. Wolff, Triple-junction solar cells with improved carrier and photon management. *Nature* **653**, 90–97 (2026).

7. Y. H. Chiang, K. Frohna, H. Salway, A. Abfalterer, L. Pan, B. Roose, M. Anaya, S. D. Stranks, Vacuum-Deposited Wide-Bandgap Perovskite for All-Perovskite Tandem Solar Cells. *ACS Energy Lett.* **8**, 2728–2737 (2023).

8. C. Liu, Y. Yang, H. Chen, J. Xu, A. Liu, A. S. R. Bati, H. Zhu, L. Grater, S. S. Hadke, C. Huang, V. K. Sangwan, T. Cai, D. Shin, L. X. Chen, M. C. Hersam, C. A. Mirkin, B. Chen, M. G. Kanatzidis, E. H. Sargent, Bimolecularly Passivated Interface Enables Efficient and Stable Inverted Perovskite Solar Cells. *Science (1979).* **384**, 330–336 (2024).
